# The Glycoprotease CpaA Secreted by Medically Relevant Acinetobacter Species Targets Multiple *O*-Linked Host Glycoproteins

**DOI:** 10.1128/mBio.02033-20

**Published:** 2020-10-06

**Authors:** M. Florencia Haurat, Nichollas E. Scott, Gisela Di Venanzio, Juvenal Lopez, Benjamin Pluvinage, Alisdair B. Boraston, Michael J. Ferracane, Mario F. Feldman

**Affiliations:** a Department of Molecular Microbiology, Washington University School of Medicine in St. Louis, St. Louis, Missouri, USA; b Department of Microbiology and Immunology, The Peter Doherty Institute for Infection and Immunity, University of Melbourne, Parkville, VIC, Australia; c Department of Biochemistry and Microbiology, University of Victoria, Victoria, British Columbia, Canada; d Department of Chemistry, University of Redlands, Redlands, California, USA; University of Georgia

**Keywords:** *Acinetobacter*, CpaA, T2SS, glycobiology, glycoprotease

## Abstract

Glycans decorate proteins and affect their biological function, including protection against proteolytic degradation. However, pathogenic, and commensal bacteria have evolved specific glycoproteases that overcome the steric impediment posed by carbohydrates, cleaving glycoproteins precisely at their glycosylation site(s). Medically relevant Acinetobacter strains employ their type II secretion system (T2SS) to secrete the glycoprotease CpaA, which contributes to virulence. Previously, CpaA was shown to cleave two *O*-linked glycoproteins, factors V and XII, leading to reduced blood coagulation. In this work, we show that CpaA cleaves a broader range of *O*-linked human glycoproteins, including several glycoproteins involved in complement activation, such as CD55 and CD46. However, only CD55 was removed from the cell surface, while CD46 remained unaltered during the Acinetobacter nosocomialis infection assay. We show that CpaA has a unique consensus target sequence that consists of a glycosylated serine or threonine residue after a proline residue (P-S/T), and its activity is not affected by sialic acids. Molecular modeling and mutagenesis analysis of CpaA suggest that the indole ring of Trp493 and the ring of the Pro residue in the substrate form a key interaction that contributes to CpaA sequence selectivity. Similar bacterial glycoproteases have recently gained attention as tools for proteomic analysis of human glycoproteins, and CpaA appears to be a robust and attractive new component of the glycoproteomics toolbox. Combined, our work provides insight into the function and possible application of CpaA, a member of a widespread class of broad-spectrum bacterial glycoproteases involved in host-pathogen interactions.

## Abstract (precis)

CpaA is a glycoprotease expressed by members of the Acinetobacter baumannii-calcoaceticus complex, and it is the first bona fide secreted virulence factor identified in these species. Here, we show that CpaA cleaves multiple targets precisely at *O*-glycosylation sites preceded by a Pro residue. This feature, together with the observation that sialic acid does not impact CpaA activity, makes this enzyme an attractive tool for the analysis of *O*-linked human protein for biotechnical and diagnostic purposes. Previous work identified proteins involved in blood coagulation as targets of CpaA. Our work broadens the set of targets of CpaA, pointing toward additional roles in bacterium-host interactions. We propose that CpaA belongs to an expanding class of functionally defined glycoproteases that targets multiple *O*-linked host glycoproteins.

## INTRODUCTION

Members of the Acinetobacter baumannii-calcoaceticus complex are a frequent cause of serious multidrug-resistant infections that are associated with high mortality, and they are a top priority for the research and development of new antimicrobial therapies (1, 2). However, the development of innovative therapies against these Gram-negative pathogens demands a better understanding of the virulence and resistance mechanisms critical to Acinetobacter infection. Previous work has implicated the type II secretion system (T2SS) in the pathogenesis of several Acinetobacter spp. in different infection models (3–8). The T2SS is a trans-envelope machine that mediates the transport of effector proteins from the periplasm to the extracellular milieu. First, T2SS effectors are translocated to the periplasmic space by either the general secretory pathway or the twin-arginine translocation (TAT) system (5). Once in the periplasm, the effectors fold into their tertiary/quaternary structure, a process occasionally facilitated by dedicated chaperones, and are subsequently targeted to the T2SS machinery for secretion (7). Although the T2SS machinery is conserved across Acinetobacter spp., the effector repertoire is diverse and varies from strain to strain (7, 9, 10).

Approximately 20 to 60 T2SS effectors are secreted by any given Acinetobacter strain, and these proteins are involved in lipid assimilation, serum resistance, colonization of various host tissues, antibiotic resistance, and biofilm formation (3, 4, 7, 11). Among these T2SS effectors, the zinc-metallo-endopeptidase CpaA is the best-characterized and most abundant effector secreted by several medically relevant Acinetobacter *strains* (7, 8, 12). We demonstrated that CpaA stability and secretion depend on the membrane-bound chaperone CpaB, which is encoded adjacent to CpaA (7, 8). The N-terminal transmembrane domain of CpaB anchors the protein to the membrane, whereas its C-terminal domain interacts directly with CpaA in the periplasm (8). Removal of the CpaB transmembrane domain results in secretion of the CpaA-CpaB complex (8). Structural studies confirmed that CpaA and CpaB strongly interact in a 1:1 ratio via a novel protease-chaperone arrangement in which CpaA surrounds CpaB (8, 13). This unusual configuration was not observed in other previously characterized T2SS chaperone/effector pairs (14). An additional interesting feature observed in the cocrystal structure is that the CpaB C-terminal tail blocks access to the CpaA catalytic site (13).

In Acinetobacter nosocomialis M2, deletion of *cpaA* results in virulence defects comparable to those observed in a T2SS mutant strain (8). Importantly, using a murine pneumonia model, we demonstrated that CpaA plays a crucial role in dissemination to the spleen (8). The link between CpaA and dissemination within the host is further supported by *in vitro* data showing that CpaA is able to cleave human factor V (fV) and factor XII (fXII), thus interfering with blood coagulation (12, 15). Interestingly, CpaA cleaves fXII at two *O*-glycosylation sites in its proline-rich region, between Pro_279_Thr_280_ and Pro_308_Thr_309_, with both Thr residues being *O* glycosylated, though glycans are known to protect proteins from degradation (15). Moreover, glycosylation is required for CpaA activity, as deglycosylation of fXII abrogates CpaA activity (15). However, the full scope of CpaA substrates and the basis for their recognition by CpaA remain poorly understood.

Few glycoprotein-targeting zinc metallo-endopeptidases have been structurally characterized. They belong to either the metzincin or gluzincin protease superfamilies (16, 17). Metzincin proteases have a characteristic extended zinc-binding motif (HEXXHXXGXXH) and a conserved Met turn, whereas gluzincin proteases have a glutamate residue in the zinc-binding motif (HEXXHE) (18). We previously determined the X-ray crystal structure of CpaA (13). Key features of its catalytic domain (an α+β fold, an extended zinc-binding motif, and a conserved Met turn) revealed that CpaA belongs to the metzincin protease superfamily (13, 19). In addition to its catalytic domain, CpaA possesses four very similar β-sheet tandem repeats, all of which share a similar Ig-like folds and resemble glycan-binding domains (13, 15). Interestingly, these repeats share fold similarities to a domain present in the metzincin protease StcE, a secreted glycoprotease from enterohemorrhagic Escherichia coli (EHEC), though their detailed sequence and structural similarities are very limited (13, 20). StcE is also a T2SS-secreted protease known to target highly *O*-glycosylated epithelial substrates, such as CD55 (16, 17, 21–23).

Given these reported structural similarities, we hypothesized that CpaA could target a broader number of glycosylated host proteins. In this work, we combined biochemistry, mass spectrometry (MS), molecular modeling and *in vivo* assays to demonstrate that CpaA targets multiple *O*-linked human glycoproteins. This work furthers our understanding of the interaction of CpaA with its targets and provides valuable insight into Acinetobacter pathobiology. Furthermore, we show that CpaA has a possible biotechnological application as a tool for glycoproteomics.

## RESULTS

### CpaA is a broad-spectrum glycoprotease.

**FIG 1:**
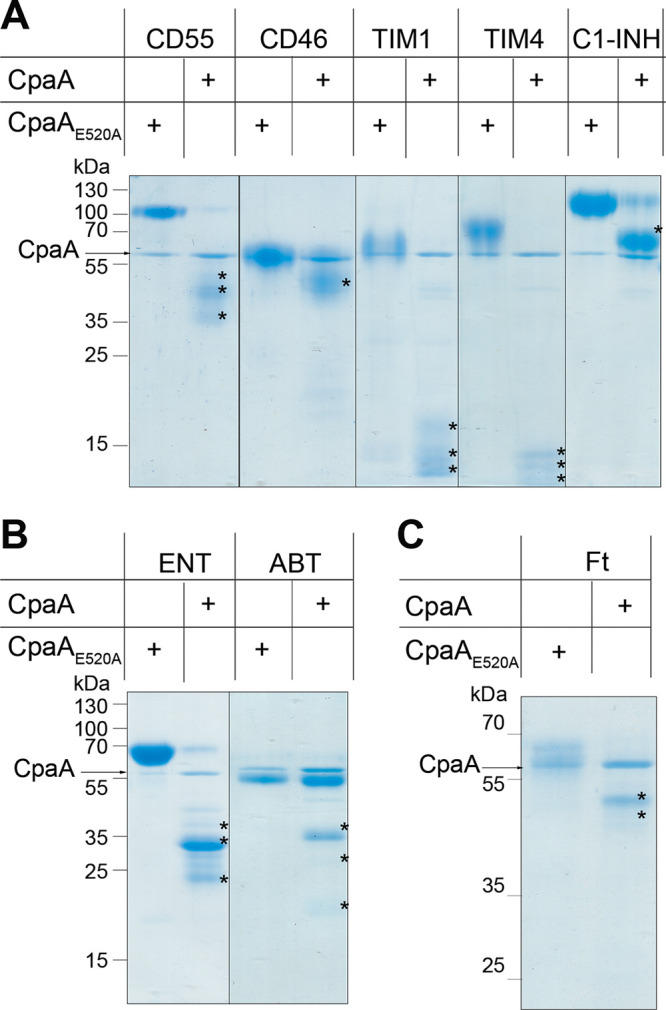
CpaA targets multiple proteins *in vitro*. Purified mucins (A), mucin-like proteins (B), and fetuin (C) were treated with purified CpaA and catalytically inactive CpaA (CpaA_E520A_) (∼55 kDa). Samples were separated by SDS-PAGE. Digestion products are indicated by asterisks. These data are representative of at least 3 independent experiments. C1-INH, C1 esterase inhibitor; TIM, T-cell immunoglobulin and mucin domain; ENT, etanercept; ABT, abatacept; Ft, bovine fetuin.

Previous reports indicated that CpaA targets coagulation-related glycoproteins, such as fV and fXII (12, 15). CpaA cleaves the fXII mucin-like region at two sites (15). Mucins are a family of high-molecular-weight glycoproteins composed of Pro/Thr/Ser-rich domains that are heavily decorated with long oligosaccharides (24, 25). While glycosylation is protein dependent, the average mucin is ∼50% *O*-linked glycan by mass, and these glycans tend to be very heterogeneous as a result of unique extensions and branching of the core structure (24, 25). Glycoproteases targeting mucins show a wide variability in terms of substrate preferences, even among members of the same family, and therefore, prediction of their targets is not possible (16, 21–23, 26–29). To assess whether CpaA can cleave other glycoproteins, we expressed and purified CpaA and its catalytically inactive point mutant (E520A) as C-terminally His-tagged proteins in A. nosocomialis M2, as was previously done (8, 13). CpaA and CpaA_E520A_ were incubated overnight with several medically relevant and commercially available glycoproteins (CD55, CD46, TIM1, TIM4, and C1 esterase inhibitor [C1-INH]), for which their glycosylation is at least partially characterized. Mucins run as large and diffuse bands as a result of their heterogeneity in size and glycosylation. Remarkably, CpaA, but not catalytically inactive CpaA_E520A_, cleaved all the tested mucin glycoproteins, as observed by gel shifts to lower molecular weight (Fig. 1A; degradation products are indicated with asterisks). We also tested the ability of CpaA to cleave two well-characterized recombinant therapeutic glycoproteins, etanercept (Enbrel) and abatacept (Orencia). These fusion proteins are highly glycosylated proteins purified from mammalian cells and contain mucin-like domains in their structures (30–32). Once again, CpaA, but not CpaA_E520A_, cleaved both recombinant glycoproteins, as observed in Fig. 1B (degradation products are indicated with asterisks).

### CpaA specifically targets O-linked glycoproteins, and its activity is unaffected by sialic acid.

**FIG 2:**
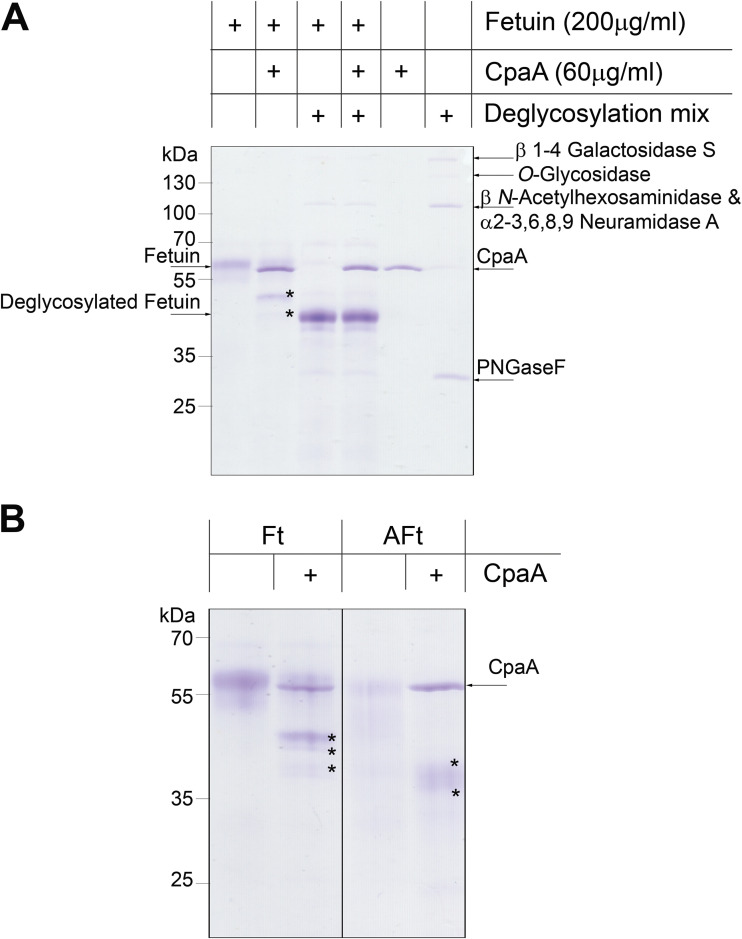
Fetuin glycosylation is required for CpaA activity. (A) Fetuin and fetuin treated with a deglycosylation mix were incubated with CpaA. Digestion products (indicated with asterisks) are observed only for fetuin. (B) The presence of sialic acid does not affect CpaA activity. Ft, bovine fetuin; Aft, asialofetuin.

The activity of glycoproteases is differentially impacted by many factors, such as *O*-glycosylation density and glycan composition. For instance, StcE and TagA, a secreted glycoprotease from Vibrio cholerae, uniquely cleave heavily glycosylated proteins, such as mucins, but they cannot digest less *O*-glycosylated proteins, such as fetuin (Ft). Fetuin, also known as alpha-2-HS-glycoprotein, is decorated with up to five *O*-linked sialylated glycans and is often used as a model protein for glycosylation-related studies (22, 26, 33). In contrast, the activity of other glycoproteases, such as the gluzincin IMPa from Pseudomonas aeruginosa, is unaffected by the degree of *O* glycosylation (16). Like IMPa, CpaA cleaved fetuin (Fig. 1C) but not enzymatically deglycosylated fetuin (Fig. 2A). The requirement of glycosylation for CpaA activity is also supported by the observation that RNase A and bovine serum albumin (BSA) were not digested by CpaA (Fig. S1A). Furthermore, our results are in agreement with those of Waack et al., who showed that enzymatically deglycosylated fXII was not cleaved by CpaA (15).

10.1128/mBio.02033-20.3 FIG S1 CpaA has substrate preferences. (A) CpaA does not cleave either exclusively *N*-glycosylated protein RNaseB or unglycosylated proteins such as RNaseA and BSA. RNase A contains traces of RNase B. RNase B, RNase A, and BSA were incubated with and without purified CpaA. Samples were separated by SDS-PAGE. No digestion products were observed. (B) CpaA cannot cleave a protein lacking a Pro residue at the P1 position. Human erythropoietin (EPO) was incubated with CpaA and catalytically inactive CpaA (CpaAE520A). No degradation of EPO was observed. This result supports the essentiality of the Pro residue preceding the *O*-glycosylation site. Download FIG S1, PDF file, 1.5 MB. Copyright © 2020 Haurat et al. 2020 Haurat et al. This content is distributed under the terms of the Creative Commons Attribution 4.0 International license.

The activity of various glycoproteases can be dependent on, inhibited by, or unaffected by the presence of sialic acid, a negatively charged 9-C sugar with key biological functions (34). For example, glycoproteases from Mannheimia haemolytica and Clostridium perfringens require sialic acid for activity, whereas other glycoproteases are inhibited by this sugar (16, 35, 36). StcE is an example of a glycoprotease that is indifferent to sialic acid (22). Similarly to StcE, CpaA efficiently digested asialofetuin (AFt), a variant of fetuin lacking only the sialic acid moieties (Fig. 2B), indicating that CpaA glycoprotease activity is sialic acid independent.

All of the CpaA targets tested so far were simultaneously *N*- and *O*-glycosylated proteins. To test whether CpaA can cleave *N*-linked glycoproteins, we incubated CpaA with RNaseB, a glycoprotein modified only with *N*-linked glycans. No proteolytic activity was observed with RNaseB as a substrate (Fig. S1). Together, these results indicate that CpaA possesses broad substrate specificity, being able to cleave multiple *O*-glycosylated proteins in addition to fV and fXII, and that CpaA activity is unaffected by sialylation.

### CpaA cleaves between Pro and a glycosylated Ser/Thr.

**FIG 3:**
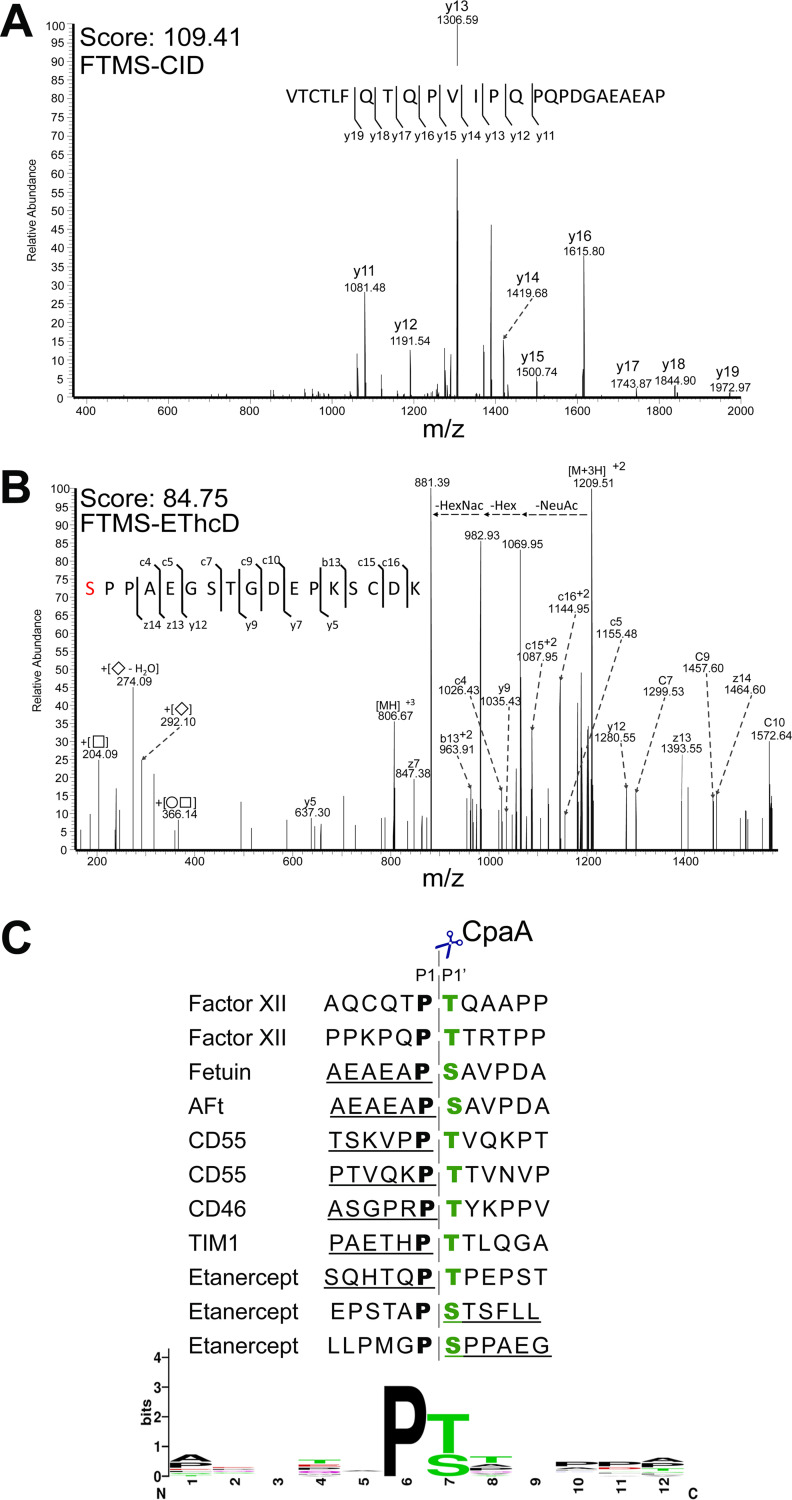
CpaA cleaves between Pro and a glycosylated Ser/Thr. Mass spectra of the nontryptic fragment of fetuin (A) and etanercept (B) are shown. The glycosylated Ser residue, indicated in red, is modified with HexNAcHexNeuAc. (C) Sequences of the CpaA-dependent cleavage products were used as WebLogo inputs (weblogo.berkeley.edu). Factor XII cleavage sites were previously reported (15). The underlined peptide sequences were detected by mass spectrometry. The dashed line indicates the CpaA cleavage site. HexNAc, *N*-acetyl hexosamine; Hex, hexose; NeuAc, *N*-acetyl neuraminic acid.

We employed an MS approach to gain insight into the common molecular features that dictate CpaA substrate recognition. We excised the gel pieces containing the proteolysis products indicated with asterisks in Fig. 1, treated them with trypsin, and subsequently subjected them to MS analysis, as done previously (37). For most of the proteins, with the exceptions of TIM4 and C1-INH, we identified nontryptic peptides, resulting from CpaA activity (Fig. 3 and Fig. S2). These peptides contained an invariant C-terminal Pro residue (P1 position) (Fig. 3A and Fig. S2A, C, and D), which in the full-length protein sequences is always adjacent to a glycosylated Ser or Thr (S/T*, where the asterisk indicates a glycosylated amino acid) (Fig. 3C). We also identified nontryptic glycopeptides derived from etanercept that contained a glycosylated N-terminal Ser (Fig. 3B and Fig. S2B). The peptides were glycosylated with either *N*-acetyl hexose-hexose-*N*-acetyl neuraminic acid (HexNAc-Hex-Neu) or HexNAc-Hex moieties (Fig. 3B and Fig. S2B), reinforcing the concept that CpaA activity is indifferent to the presence of sialic acid. The CpaA-dependent cleavage products, including those of fXII (previously reported), were used as WebLogo inputs (Fig. 3C) (15, 38). This analysis revealed that CpaA has a distinct peptide consensus sequence, P-S/T*, where cleavage occurred before the glycosylated Ser/Thr residue. As seen in Fig. 3C, the C-terminal Pro residue preceding the *O*-glycosylation site is likely a strict requirement for CpaA targeting. Human erythropoietin (EPO) contains the same Ser-bound oligosaccharide as fetuin but has an Ala residue preceding the glycosylation site. This protein was not cleaved by CpaA (Fig. S1B), further supporting the essentiality of the Pro residue preceding the *O*-glycosylation site.

10.1128/mBio.02033-20.4 FIG S2 CpaA cleaves between Pro and a glycosylated Ser/Thr. Mass spectrometry spectra of the nontryptic fragment of CD55 (A), which is modified with HexNAcHexNeuAc and etanercept (B), which is modified with three glycans: two HexNAcHexNeuAc and one HexNAcHex. (C) Etanercept. (D) CD46. Glycosylated residues are indicted in red. HexNAc, *N*-acetyl hexosamine; Hex, hexose; NeuAc, *N*-acetylneuraminic acid. Download FIG S2, TIF file, 1.1 MB. Copyright © 2020 Haurat et al. 2020 Haurat et al. This content is distributed under the terms of the Creative Commons Attribution 4.0 International license.

### Mammalian glycan array screening.

The recombinant proteins tested in this study were all expressed and purified from HEK293 cells. This cell line generates proteins *O*-glycosylated with the disaccharide structure galactose β1-3 *N*-acetylgalactosamine (Galβ1-3GalNAc), also known as core I, as well as mono- and disialylated core I and core II (Galβ1-3[GlcNAcβ1-6]GalNAc) structures (39). Other glycan cores have more complex (branched) structures that may or may not be accommodated by CpaA (40). To explore this further, we employed a mammalian glycan array screening assay (performed at the Consortium for Functional Glycomics) to test if CpaA can directly bind various glycans. However, we did not detect any significant peak indicative of binding for any glycan in the array when employing two protein concentrations (5 μg/ml and 50 μg/ml) of either CpaA or its catalytically inactive mutant CpaA_E520A_, (Fig. S3). A similar lack of binding has been reported for other glycoproteases, which may be due to low-affinity or transient interactions with the glycans (41). It is noteworthy that none of the glycans in the glycan array are conjugated to P-S/T sequences. Considering our previous results, it is likely that recognition by CpaA is dependent on a combination of both protein sequence and glycan structure adopted in the array.

10.1128/mBio.02033-20.5 FIG S3 Glycan array assay. We did not detect any significant peak indicative of any glycan in the array when employing two protein concentrations (5 μg/ml and 50 μg/ml) of either CpaA (A) or its catalytically inactive mutant CpaA_E520A_ (B). Binding is considered significant at values above 1,000 relative fluorescence units (RFU). Download FIG S3, TIF file, 0.5 MB. Copyright © 2020 Haurat et al. 2020 Haurat et al. This content is distributed under the terms of the Creative Commons Attribution 4.0 International license.

### CD55 is removed from epithelial surfaces by secreted CpaA.

**FIG 4:**
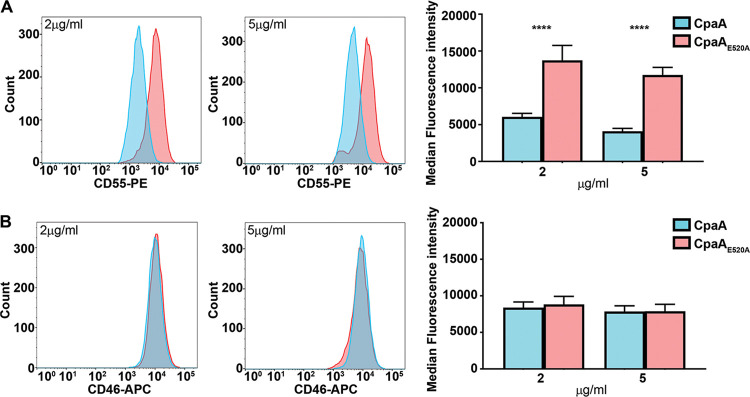
Purified CpaA cleaves CD55 but not CD46 from HeLa cell surfaces. Surface levels of CD55 (A) and CD46 (B) were measured by flow cytometry on HeLa cells. Cells were incubated with two different concentrations (2 μg/ml and 5 μg/ml) of purified CpaA and CpaA_E520A_ for 1 h. Left and middle panels are representative flow cytometry histograms. Right panels show the medians and standard deviations (SD) for three biological repeats. ****, *P* < 0.0001 (two-way ANOVA with Sidak's multiple-comparison test).

Our previous *in vitro* results expanded the known CpaA substrates to include human glycoproteins beyond those involved in blood coagulation (Fig. 1A). To gain insight about CpaA activity in the context of an infection, we tested whether CpaA directly cleaves surface exposed *O*-glycoproteins. Of all the glycoproteins tested *in vitro*, CD55 and CD46 are highly expressed cell surface *O*-glycoproteins in HeLa cells (42). Thus, HeLa cells were treated with purified CpaA and CpaA_E520A_, and the levels of CD55 and CD46 bound to the cell surface were quantified by flow cytometry (Fig. 4). At the two protein concentrations tested, cells treated with CpaA displayed a reduced amount of CD55 on their surfaces compared to those treated with CpaA_E520A_ (Fig. 4A). In contrast, although CpaA was able to cleave CD46 in our *in vitro* assay (Fig. 1A), levels of cell surface-exposed CD46 remained unaltered after CpaA treatment, independent of the protein concentration used (Fig. 4B).

**FIG 5:**
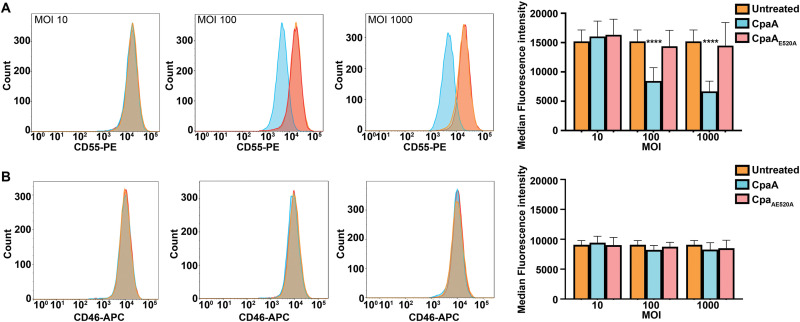
Secreted CpaA cleaves CD55 from HeLa cell surfaces. Surface levels of (A) CD55 and (B) CD46 were measured by flow cytometry on HeLa cells. Cells were incubated with three different MOI (10, 100, and 1,000) of A. nosocomialis M2 secreting either CpaA or CpaA_E520A_ for 2.5 h. Representative flow cytometry histograms are shown. Right panels show the medians and SD for three biological repeats. ****, *P* < 0.0001 (two-way ANOVA with Dunnett's multiple-comparison test).

Next, we infected HeLa cells with A. nosocomialis M2 expressing CpaA and CpaA_E520A_ at three different multiplicities of infection (MOI) (10, 100, and 1,000) and used flow cytometry analysis to quantify the levels of cell surface exposed CD55 and CD46 postinfection (Fig. 5). CD55 and CD46 levels remained unchanged when cells were infected with A. nosocomialis M2 secreting CpaA_E520A_, indicating that A. nosocomialis M2 does not secrete any other proteases targeting either glycoprotein (Fig. 5). In agreement with our previous results, secreted CpaA cleaved CD55 but not CD46 from the cell surface (Fig. 5). The CD46 protein used in the *in vitro* assay (Fig. 1A) was expressed and purified from HEK293 cells. It is well known that glycosylation patterns differ between cell lines (43); thus, it is possible that different protein glycosylation patterns impact CpaA activity. To address this, we repeated the experiment infecting HEK293 cells. As observed with HeLa cells, secreted CpaA digested CD55 but not CD46 (Fig. S4), indicating that potential differences in glycosylation between these two cells lines do not account for these discrepancies. Importantly, an MOI of 100 was sufficient to detect cleavage of CD55 from HeLa cells, and increasing the MOI to 1,000 did not boost CD55 cleavage by CpaA (Fig. 5A). The remaining CD55 (and perhaps CD46) may be associated with proteins/ligands that prevent CpaA activity. We conclude that A. nosocomialis secretes physiological levels of CpaA that can digest host surface-exposed proteins during infection.

10.1128/mBio.02033-20.6 FIG S4 Secreted CpaA cleaves CD55 from HEK293 cell surfaces. Surface level(B) of CD55 (A) and CD46 (B) were measured by flow cytometry on HEK293 cells. Cells were incubated of A. nosocomialis M2 secreting either CpaA or CpaA_E520A_ at an MOI of 100 for 2.5 h. Representative flow cytometry histograms are shown. Right panels show medians and SD for three biological repeats. *, *P* < 0.02 (two-way ANOVA with Tukey's multiple-comparison test). Download FIG S4, PDF file, 2.1 MB. Copyright © 2020 Haurat et al. 2020 Haurat et al. This content is distributed under the terms of the Creative Commons Attribution 4.0 International license.

### Molecular modeling identifies a putative mode of binding of glycopeptides to CpaA.

**FIG 6:**
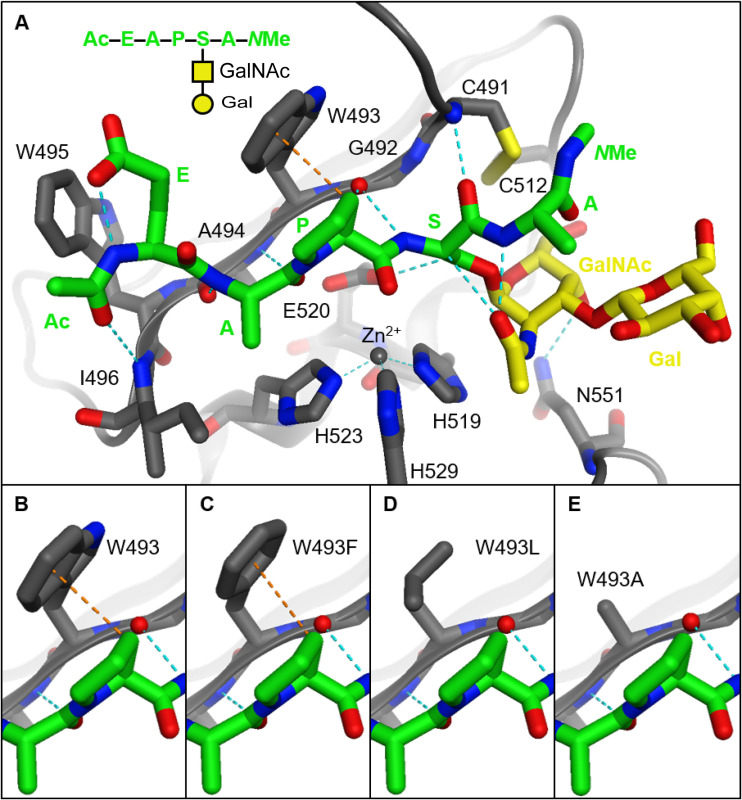
Structure of a fetuin-based model glycopeptide substrate docked in models of CpaA and related mutants. (A) The enzyme (gray) and the substrate’s peptide portion (green) form hydrogen bonds (dashed blue lines) between their backbones similar to an antiparallel beta sheet. The catalytic histidine residues (gray sticks) and zinc ion (gray sphere) hydrolyze the amide bond between the substrate’s proline (P1) and glycosylated serine (P1′) residues. The GalNAc moiety (yellow sticks) forms hydrogen bonds with the substrate and the enzyme, whereas the Gal moiety (yellow sticks) is exposed to solvent. (B to E) The tryptophan residue of the native enzyme can form an H-π interaction (dashed orange lines) with the substrate. This is interaction is weakened when this residue is mutated to phenylalanine and nonexistent with aliphatic residues.

We previously determined the X-ray cocrystal structure of the CpaA-CpaB complex (13), and others have obtained crystal structures of related metzincin enzymes with peptide- and peptidomimetic-based ligands (Table 1) (44, 45). X-ray structures of the related gluzincin glycopeptidases BT4244 (Bacteroides thetaiotaomicron), IMPa, and ZmpB (Clostridium perfringens ATCC 13124) have also been determined with bound glyco-amino acid and glycopeptide ligands (16). Notably, these gluzincin enzymes cleave fetuin, asialofetuin, and related synthetic glycopeptides to various degrees (Table 1). Thus, to better understand binding between CpaA and glycopeptide substrates, we performed docking experiments between a CpaA model and three different glycoforms of a fetuin-based fragment peptide (Ac–EAPSA–*N*-methyl [*N*-Me], where S is glycosylated). One of the glycoforms lacks the sialic acid moiety (Fig. 6 and Fig. S5A), while the other two are sialylated at two different position of the Galβ1-3GalNAc core (Fig. S5B and C). The peptide portions of the docked species were able to contact the catalytic zinc ion while forming an antiparallel β-sheet with a β-strand of the active site (Fig. 6A), findings that are consistent with aforementioned metzincin crystal structures as well as docking experiments with StcE (16, 22). In the docked structures, the consensus P1 proline residue lies adjacent to W493 and is somewhat solvent exposed (Fig. 6B). An H-π interaction was observed between a proline beta hydrogen and the tryptophan indole ring (Fig. 6B). These studies suggest that CpaA selectivity may be the result of W493 (i) forming a potential H-π interaction with the prolyl ring, (ii) minimizing the prolyl residue’s exposure to solvent, and/or (iii) sterically holding the substrate in the active site.

**TABLE 1:** Summary of the biochemical and structural features of known *O*-glycoproteases

Glycoprotease	Source	Secretion	MEROPS	Family	Structural domain organization	Target mucins	Target fetuin	Sialic acid requirement	Recognition site	Reference(s)
CpaA	A spp.	T2SS	M72	Metzincin	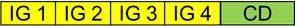	Yes	Yes	Indifferent	P-S/T*	This study; 13
StcE	EHEC	T2SS	M66	Metzincin	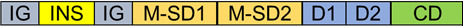	Yes	No	Indifferent	S/T*X-S/T	17, 22, 33
BT_4244	Bt	Extracellular lipoprotein	M60	Gluzincin	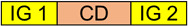	Yes	No	No sialic acid	T/S-T/S*	16
IMPa	Pa	T2SS	M88	Gluzincin	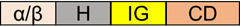	Yes	Yes	Its removal enhances activity	ND	12, 29, 68
ZmpB	Cf	SP	M60	Gluzincin	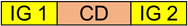	Yes	Yes	Required	TE-T/ST/S	16
Pic	Ec	T5SS	S6	SPATE	Structure undetermined	Yes	No	Required	T/S-S/T*	51, 69, 70
ZmpC	Sp	Sortase	M26		Structure undetermined	Yes	Yes	Indifferent	T*XXX-X	51
AMUC_0627	Am	SP, extracellular, unknown	M60	Gluzincin	Structure undetermined	Yes	No	Its removal enhances activity	S/T*-S/T*	51
AMUC_0908	Am	SP, extracellular, unknown	M60	Gluzincin	Structure undetermined	Yes	No	Indifferent	T/S-T/S*	51
AMUC_1514	Am	Extracellular, unknown	M60	Gluzincin	Structure undetermined	Yes	No	No sialic acid	T/S-T/S*	51
TagA	Vc	T2SS	M66	Metzincin	Structure undetermined	Yes	No	ND	TE-T/ST/S	51
SslE/YghJ	Ec/Sf	T2SS	M98	Gluzincin	Structure undetermined	Yes	ND	ND	ND	28, 71
O-sialoglycoprotease	Mh	Extracellular, unknown	M22	NA	Structure undetermined	Yes	No	Required	ND	70, 72

ND, not determined; NA, not applicable; CD, catalytic domain; IG, Ig-like domain; INS, insertion domain; M-SD, metalloprotease module; D, disordered region; α/β, α/β domain; H, helix bundle linker; A, Acinetobacter; Bt, Bacteroides thetaiotaomicron; Pa, Pseudomonas aeruginosa; Cf, Clostridium perfringens; Ec, Escherichia coli; Am, Akkermansia muciniphila; Vc, Vibrio cholerae; Sf, Shigella flexneri; Mh, Mannheimia haemolytica. Asterisk indicates that the amino acid residue is glycosylated.

10.1128/mBio.02033-20.7 FIG S5 Comparison of CpaA-glycopeptide docked structures and related gluzincin X-ray crystal structures. (A to C) The glycans of the docked fetuin-based model substrates form few contacts (dashed blue lines) with CpaA (gray), though a contact between the 4-OH of the GalNAc moiety and side chain of N551 is consistently observed. (D to F) The related gluzincin enzymes BT4244 (orange), IMPa (tan), and ZmpB (brown) form contacts with the GalNAc moieties using the side chains of conserved tryptophan and arginine residues. Further contacts are observed between IMPa and ZmpB and the additional residues of their respective glycans. Download FIG S5, PDF file, 0.2 MB. Copyright © 2020 Haurat et al. 2020 Haurat et al. This content is distributed under the terms of the Creative Commons Attribution 4.0 International license.

In the docked structures, the glycan moieties were found to form few interactions with CpaA and the substrate peptide. The acetyl group of the GalNAc moieties all formed hydrogen bonds with the peptide backbone (Fig. 6A). Similar contacts were observed in docking studies using *O*-GalNAc-ylated peptides and StcE (22). Such contacts have been shown to affect the conformation of mucin-like glycopeptides (46), and they may bias substrates into an extended conformation that would be more easily recognized by the enzyme. We also found that the 4-OH of the GalNAc moieties formed hydrogen bonds with the side chain amide of N551 (Fig. 6A and Fig. S5A to C). This interaction would likely endow the enzyme with selectivity for peptides modified with GalNAc at P1′. Interestingly, the side chain of this residue occupies space similar to that of the side chains of tryptophan residues that are conserved in the related gluzincin enzymes BT4244, IMPa, and ZmpB (Fig. S5D and E). In the gluzincin enzymes, the indole nitrogens instead hydrogen bond to the acetyl groups of GalNAc ligands. Such a contact may be formed between CpaA and its substrates if minor conformational changes occur. In either case, N551 appears to be the main residue recognizing the unique features of the GalNAc moiety, as the other residues flanking the glycans are mostly small and nonpolar. The related gluzincin enzymes, on the other hand, form several contacts between polar side chains and their GalNAc groups.

Larger, linear glycans project subsequent sugar residues into solvent; these groups are not predicted to interact with CpaA. Branched glycans are not well accommodated by the enzyme, as the disulfide bond adjacent to the active site limits the flexibility of the enzyme and the branched sugar’s ability to bind. Conversely, both IMPa and ZmpB form interactions with additional sugars of their corresponding glycans. Still, only IMPa demonstrated binding activity in the mammalian glycan array screen (16), which may explain why no hits were found in the same screen with CpaA. Collectively, our docking studies indicate that CpaA interacts with both peptide and glycan components of the substrate and identify residue W493 as a potential mediator of the interaction between CpaA and the Pro residue of its substrate.

### Effect of W493 on CpaA specific activity.

Docking studies suggest an H-π interaction between the indole ring (of W493) of CpaA and a beta hydrogen of Pro residue (at the P1 position) of the substrate (Fig. 6B). While other substrate residues can form this contact with W493, the unique rigidity and geometry of Pro allow it to optimally present its beta hydrogen to the indole ring of W493. Thus, we hypothesized that W493 of CpaA plays a critical role in CpaA selectivity. Indeed, our molecular docking studies indicate a weaker H-π interaction when peptides were docked into a mutant W493F model (Fig. 6C). The modeled W493L and W493A mutants have aliphatic residues that are unable to form this interaction with the substrate (Fig. 6D and E). Moreover, all the mutant models formed fewer van der Waals contacts with the Pro residue and further exposed it to solvent.

**FIG 7:**
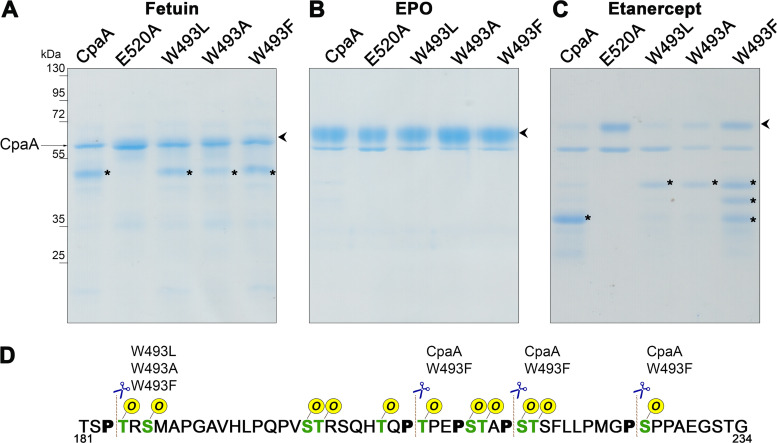
W493 affects CpaA activity. Fetuin (A), EPO (B), and etanercept (C) were incubated with purified CpaA and the different CpaA mutants (∼55 kDa). Samples were separated by SDS-PAGE, and gels were stained with InstantBlue. Digestion products are indicated by asterisks. Undigested products are indicated by arrowheads. These data are representative of at least 3 independent experiments. (D) Fragment of the etanercept protein sequence. MS analysis of proteins bands allowed the identification of the sites preferentially cleaved by the four CpaA variants. All the known *O*-glycosylation sites are in green, and, the Ps next to the glycosites are in bold. The dashed lines indicate the cleavage sites of CpaA and the different point mutants.

To complement our molecular modeling experiments, we tested the effect of these mutations on CpaA activity. All CpaA point mutation variants were expressed and secreted at levels similar to those of CpaA and CpaA_E520A_, and no degradation products of CpaA were observed in the whole-cell fractions of the CpaA variants (Fig. S6A and B). CpaA_E520A_ was included as a negative control for CpaA activity. We purified all His-tagged CpaA variants and determined their *in vitro* activities against various substrates (Fig. 7A and Fig. S6C and S7). The different mutations affected CpaA efficiency and site recognition in a substrate-specific manner. All mutants except CpaA_E520A_ were able to cleave fetuin, yielding a similar cleavage pattern (Fig. 7A). However, CpaAW493A cleaved fetuin with less efficiency. None of the CpaA variants were able to cleave EPO, further highlighting the essentiality of a Pro residue at P1 for targeting by CpaA (Fig. 7B). Treatment of CD55 and C1-INH with the CpaA mutants revealed that all variants are less active, as shown by an increase in the amounts of undigested substrate. Additional faint bands were observed, but we were unable to determine the cleavage site using MS analysis (Fig. S7).

10.1128/mBio.02033-20.8 FIG S6 CpaA point mutations in W493 are stable and secreted to levels similar to those of wild-type CpaA. (A) Western blot analyses shown that CpaA was similarly expressed in A. nosocomialis whole cells. No degradation fragments were observed. (B) The secretion of CpaA variants to the supernatant was not affected. RNA polymerase was used as loading and lysis controls for whole cell and supernatant samples, respectively. (C) The quality of the purification of CpaA mutant proteins was assessed by SDS-PAGE; gels were stained with InstantBlue. Download FIG S6, TIF file, 2.5 MB. Copyright © 2020 Haurat et al. 2020 Haurat et al. This content is distributed under the terms of the Creative Commons Attribution 4.0 International license.

10.1128/mBio.02033-20.9 FIG S7 W493 affects CpaA activity. CD55 (A) and C1-INH (B) were incubated with purified CpaA and the different CpaA mutants (∼55 kDa). Samples were separated by SDS-PAGE, and gels were stained with InstantBlue. Digestion products are indicated by asterisks. These data are representative of at least 3 independent experiments. Download FIG S7, TIF file, 1.2 MB. Copyright © 2020 Haurat et al. 2020 Haurat et al. This content is distributed under the terms of the Creative Commons Attribution 4.0 International license.

We previously identified three cleavage sites for CpaA on the mid-region of etanercept, Pro_207_Thr_208_, Pro_215_Ser_216_, and Pro_225_Ser_226_ (Fig. 3B and Fig. S2). Cleavage by CpaA generates two fragments of similar molecular weights that comigrate as a single band of about ∼36 kDa in SDS-PAGE (Fig. 1A and 7C). Notably, digestion of etanercept by CpaAW493A and W493L generated a major product of ∼45 kDa instead (Fig. 7C). CpaAW493F produced three bands that migrate as ∼36-, 40-, and 45-kDa fragments, respectively (Fig. 7C). MS analysis of these bands allowed the identification of the sites preferentially cleaved by the three CpaA variants (Fig. 7D). The unique ∼45-kDa band results from cleavage at the Pro_183_Thr_184_ site, which is not a preferred site for wild-type CpaA. Unlike the other cleavage sites, the Pro_183_Thr_184_ site is in an area of low glycosylation of etanercept. Interestingly CpaAW493A and W493L variants were unable to cleave etanercept in the high-glycosylation-density region, which could explain the low activity against other mucin targets. Taken together, our data suggest that W493, although is not essential for activity, plays a role in CpaA substrate selectivity by interacting with the Pro residue of its target protein.

### CpaA belongs to an expanding, functionally defined class of surface-exposed and secreted glycoproteases.

Mounting evidence indicates that both commensal and pathogenic species produce and secrete glycoproteases to modulate adherence, penetrate the inner mucus layer, or evade the host immune response (18, 21, 47). For example, StcE contributes to immune evasion during EHEC infection by preventing immune cells from moving to the sites of infection (20, 23, 48, 49). Additionally, StcE activity against mucins promotes access of EHEC to epithelial cells, which assists host cell colonization (20, 50). P. aeruginosa produces IMPa, which cleaves the macrophage surface protein CD44, inhibiting phagocytosis (29). IMPa also cleaves P-selectin glycoprotein ligand 1 (PSGL-1), helping the bacterium to escape neutrophil attack (29). In Vibrio cholerae, secreted TagA targets host cell surface glycoproteins, modulating bacterial attachment during infection (26). These examples are indicative of the pivotal role of glycoproteases in modulating host-pathogen interactions by targeting various host proteins. Despite their relevance, only a relatively small number of bacterial glycoproteases have been biochemically characterized to various extents (Table 1). These enzymes are commonly encoded by bacteria isolated from mucin-rich environments, such the human gut and lungs. These glycoproteases differ in their secretion mechanisms, protease class, domain organization, catalytic site, and recognized targets. Like IMPa, StcE, TagA, and SslE/YghJ, CpaA is secreted by a T2SS. CpaA, IMPa, and ZmpC display broad *O*-glycoprotease activity targeting mucins, regardless of their glycosylation density, as well as *O*-glycoproteins with low *O*-glycan chain density (fetuin). On the other hand, StcE can cleave only mucins with long mucin-like regions (such as CD43 and CD55) or short mucin-like regions (such as C1-INH). In contrast, other glycoproteases can target only a subset of mucins. For example, TagA requires more extensive densely glycosylated regions, whereas SslE/YghJ can digest major mucins such as MUC2 and MUC3, but it is inactive against mucins like CD43 and bovine submaxillary mucin (9, 23, 37, 51).

Due to low amino acid sequence conservation, it is not possible to differentiate proteases from glycoproteases solely on the basis of primary amino acid sequence. However, structural analyses revealed the presence of Ig-fold domains in all these enzymes (Table 1, domains in yellow). Moreover, several factors impact substrate targeting by glycoprotease, including glycan chain identity and density, as well as amino acid composition (Table 1). Although some target motifs are known, the molecular bases for the recognition of specific target sequences remain poorly understood. Thus, even if structural analyses identify putative glycan-binding domains in a protein of interest, it is difficult to predict the specific substrates targeted by the putative glycoprotease. Together with the functional analysis, these studies define a functional class of secreted *O*-glycoproteases that mediate host-pathogen and host-commensal interactions.

## DISCUSSION

Glycan chains decorate proteins to accomplish many different functions. One important role of glycosylation is the protection of proteins against proteolytic degradation. However, there is growing evidence that bacterial pathogens and commensals have evolved specific proteases that overcome the steric impediment posed by carbohydrates and indeed use glycans as recognition determinants to cleave glycoproteins right at the glycosylation site. In this work, we functionally characterized CpaA, a metzincin glycoprotease and T2SS-secreted virulence factor of several medically relevant Acinetobacter strains (4, 7, 8, 12). Previous work identified the blood coagulation proteins fV and fXII as targets of CpaA, suggesting a potential role in dissemination by interfering with the intrinsic coagulation pathway (4, 8, 12). The present work expands the known targets of CpaA and indicates that CpaA is a broad-spectrum enzyme with the ability to cleave various *O*-linked human glycoproteins. Our MS analysis of proteolytic fragments resulting from glycoprotein treatment with CpaA revealed that CpaA has a consensus target sequence consisting of a Pro residue followed by a glycosylated Ser or Thr (P-S/T), which is unprecedented for bacterial glycoproteases. Unlike other secreted glycoproteases, CpaA activity is not affected by sialic acid and is not restricted to highly *O*-glycosylated proteins (mucins). Indeed, CpaA also cleaves sparsely *O*-glycosylated proteins, such as fetuin. Although broad-spectrum secreted or surface-exposed glycoproteases appear to be widespread in bacteria, their identification cannot be assigned based on sequence homology, and biochemical and structural analyses are required to designate them as glycoproteases.

CpaA is composed of four very similar Ig-like domains and a catalytic domain (13). The catalytic domain located at the C terminus of CpaA exhibits all the canonical structural features of the metzincin superfamily. The four Ig-like domains are arranged in tandem, and they resemble the insertion domain of StcE, secreted by EHEC (13). These observations prompted us to further characterize CpaA activity. The StcE-specific motif S/T*-X-S/T (the asterisk denotes the glycosylation site) diverges from the P-S/T* motif recognized by CpaA (22). It is intriguing that despite recognizing different motifs, StcE and CpaA share the substrates C1-INH and CD55. Considering the different domain organization of the two proteases, it is not surprising that the proteins are classified in different metzincin subfamilies and target different proteins.

We previously showed that the substrate-binding cleft of CpaA is formed by residues from its four Ig-like domains and its catalytic domain (13). Thus, to be recognized by CpaA, the glycosylated substrate has to expose the P-S/T* peptide bond targeted for hydrolysis. It has been proposed that the interaction of the *O*-glycans with residues in the Ig-like domains (referred to as G sites by Noach et al. [16]) help to position the targeted peptide bond in the correct conformation to interact with the amino acids involved in catalysis (13, 16). Here, we show that CpaA activity is unaffected by the presence of sialic acid, indicating that the sialic acid can be accommodated inside the cleft but is not required for glycopeptide recognition. Our molecular modeling of CpaA with glycosylated substrates showed that linear glycans modified by sialic acid project this moiety away from the active site and into solvent, which supports our *in vitro* findings. Our modeling also revealed a possible interaction between the indole ring of W493 and the ring of the Pro residue in the targeted sequence. Although the digestion of glycoproteins with W493 mutants displayed substrate-specific behaviors, overall, the mutants were less active and, in some cases, exhibited a shift in glycosylation site (glycosite) preference. We propose that CpaA selectivity may, at least in part, be the result of W493 forming a potential H-π interaction with the prolyl ring, minimizing the prolyl residue’s exposure to solvent, and/or sterically holding the substrate in the active site. Further structural and biochemical studies are required to uncover the structural features that enable CpaA to target such a remarkably broad range of O-linked glycoproteins.

CpaA expression and secretion occur across several medically relevant Acinetobacter strains. Deletion of CpaA resulted in attenuation of A. nosocomialis M2 virulence in a respiratory murine infection model, playing a role in the dissemination from the lungs to the spleen (8). Previously, the only known substrates for CpaA were fV and fXII, proteins involved in blood coagulation. By digesting these proteins, CpaA increases the clotting time of human plasma (12, 15). We have now shown that CpaA can indeed cleave multiple proteins *in vitro*. Among these are several proteins involved in regulation of complement activation, including CD55 and CD46. However, only CD55 was removed from the cell surfaces, while CD46 remained unaltered during the A. nosocomialis infection assay. We hypothesize that CD46 interacts with another protein that blocks CpaA access. Thus, not all CpaA targets identified by our *in vitro* experiments are representative bona fide targets of CpaA *in vivo.* An additional role of CD55 is to act as an anti-adhesive molecule that regulates the release of neutrophils (52). Degradation of CD55 increases the retention of the neutrophils to the apical epithelial surface with the concomitant reduction of the amount of neutrophils that cross the epithelium (48, 49). CpaA expression and secretion are conserved across several medically relevant Acinetobacter strains isolated from diverse anatomical sites (8). We aligned the amino acid sequences of CpaA from A. nosocomialis M2 and several medically relevant A. baumannii strains (Fig. S8). We observed extremely high protein identity, suggesting that our findings can be extended to CpaA orthologs secreted by A. baumannii. Considering the broad-spectrum activity of CpaA and the abundance of glycosylated proteins in the human host, we propose that the physiological role of CpaA likely extends beyond interfering with the coagulation pathway or complement cascade. Further work will be required to understand the full extent of host immunomodulation by CpaA.

10.1128/mBio.02033-20.10 FIG S8 CpaA sequence is highly conserved in A. baumannii. Protein sequence alignment shows high sequence homology between CpaA from A. nosocomialis M2 and several A. baumannii strains. Multiple-protein alignment was performed using T-Coffee. Accession numbers are as follows: AN_M2, QCP65399.1; AB031, AHX84113.1; UPAB1, QCR58330.1; J15, TRY09797.1; MRSN21681, TQF20479.1; MRSN11669, TPV11181.1; MRSN14237, TPU61183.1; Ab04, AKQ30246.1; XH858, AMN01926.1; NIPH_335, ENW44339.1; OIFC098, EKL48403.1; 1419130, EXB29740.1; NIPH_601, ENW52234.1. Download FIG S8, PDF file, 0.1 MB. Copyright © 2020 Haurat et al. 2020 Haurat et al. This content is distributed under the terms of the Creative Commons Attribution 4.0 International license.

Host mucins and *O*-glycoproteins are major components of mucus, and they are ubiquitously expressed on cellular surfaces, where they act as physical barriers, receptor ligands, and mediators of intracellular signaling (53, 54). *O*-Glycoproteins and mucins lack a consensus sequence for *O*-glycosylation, and their *O*-linked glycans are highly heterogeneous in their glycan composition, numbers of residues, and linkages. However, aberrant mucin expression and glycosylation are also linked to various disease states, making mucins reliable biomarkers (54). For example, the mucin MUC1 is aberrantly expressed in the majority of cancers diagnosed each year in the United States. (53). Thus, the assessment of the mucin glycosylation status has high relevance for diagnosis of cancer and other diseases. Mucin domains are resistant to most commercially available proteases, which makes them difficult to analyze by traditional MS strategies. Bacterial glycoproteases have recently gained attention as tools for proteomic analysis of human glycoproteins (16, 22, 55). Our study shows that the broad-spectrum *O*-linked glycoprotease activity of CpaA is not affected by sialic acids. Moreover, it consistently digests any *O*-linked glycoprotein containing the P-S/T* sequence. These properties not only make CpaA a versatile enzyme modulating host-pathogen interactions but also highlight it as a robust and attractive new component of the glycoproteomics toolbox.

## MATERIALS AND METHODS

### Strains, plasmids, and growth conditions.

Bacterial strains and plasmids used in this study can be found in Table S1. E. coli Stellar and A. nosocomialis M2 cells were grown in Lennox broth (LB) at 37°C. pWH1266-based plasmids were selected with tetracycline (5 μg/ml).

10.1128/mBio.02033-20.1 TABLE S1 Strains, plasmids, and primers used in this study. Download Table S1, PDF file, 0.1 MB. Copyright © 2020 Haurat et al. 2020 Haurat et al. This content is distributed under the terms of the Creative Commons Attribution 4.0 International license.

### Generation of CpaA point mutations.

The PCR primers used for site-directed mutagenesis are listed in Table S1. To generate CpaAW493 variants, pWH-*cpaA*-*his*-*cpaB* was used as the template. PCR was carried out using Phusion DNA polymerase (Thermo Scientific). Site-directed mutagenesis was performed according to the method described by Fisher and Pei (56). Reaction products were transformed into E. coli Stellar cells and transformants were selected on LB agar supplemented with tetracycline. Mutagenesis was confirmed by DNA sequencing. The plasmids were then electroporated into the electrocompetent A. nosocomialis M2 Δ*cpaAB*::*frt* strain.

### Protein purification.

CpaA-His and its point mutation variants were purified from the supernatant of A. nosocomialis M2 (8). Briefly, A. nosocomialis M2 Δ*cpaAB*::*frt* strains carrying the pWH-based plasmids were grown in LB to mid-log phase. CpaA-His-tagged variants were purified by nickel affinity chromatography from cell-free filtered spent medium, as described before (8). Briefly, cells were pelleted at 8,000 × *g* for 10 min. Cell-free supernatants were obtained by filtration using a Nalgene Rapid-Flow filter unit (pore size, 0.2 μm) (Thermo Scientific), followed by concentration by approximately 3-fold using an Amicon Ultra-15 centrifugal filter unit (nominal molecular weight limit [NMWL], 10,000). Binding buffer (10×) was added to the cell-free supernatant prior to loading onto a nickel-nitrilotriacetic acid (Ni-NTA) agarose column (Gold Bio, St. Louis, MO) equilibrated with 1× binding buffer (50 mM NaH_2_PO_4_, 300 mM NaCl, 10 mM imidazole; pH 8.0). The column was washed with 20 column volumes (CV) of binding buffer and 10 CV of sashing buffer (50 mM NaH_2_PO_4_, 300 mM NaCl, 50 mM imidazole; pH 8.0). Proteins were eluted with elution buffer (50 mM NaH_2_PO_4_, 300 mM NaCl, 300 mM imidazole; pH 8.0). The purified proteins were concentrated, and buffer was exchanged for 20 mM HEPES, 150 mM NaCl, 50% glycerol (pH 7.4) using Amicon Ultra centrifugal filter units.

### CpaA activity assay.

Purified CpaA-His-tagged variants (60 μg/ml) were assayed in reaction buffer (20 mM HEPES [pH 7.4], 150 mM NaCl, 1 mM ZnCl_2_) containing the substrates proteins (200 μg/ml) in a final volume of 15 μl. All reaction mixtures were incubated at 37°C overnight. Reactions were monitored by SDS-PAGE, with staining with Coomassie blue or InstantBlue. Substrate proteins used in this study included CD55, CD46, TIM1, TIM4 C1-INH, erythropoietin (Sino Biological), asialofetuin (Sigma), fetuin and RNase B (New England Biolabs), etanercept (Enbrel), and abatacept (Orencia).

### Immunoblotting.

Bacterial whole-cell and supernatant samples were prepared as before (7, 8). Briefly, cultures were grown to an optical density at 600 nm (OD_600_) of 0.5, and the OD 0.5 cultures were pelleted by centrifugation and resuspended in 50 μl of Laemmli buffer for the whole-cell samples. Supernatant samples were obtained by trichloroacetic acid (TCA) precipitation of cell-free supernatants (7, 8). Protein samples were analyzed by SDS-PAGE, transferred to a nitrocellulose membrane, probed with polyclonal anti-CpaA (1:1,000) (8) and/or monoclonal anti-RNA polymerase (1:2,000; BioLegend). Western blots were probed with IRDye-conjugated secondary antibodies and visualized with an Odyssey CLx imagining system (Li-COR Biosciences, Lincoln, NE).

### Fetuin deglycosylation.

Fetuin was deglycosylated under denaturing conditions using a protein deglycosylation mix II kit (New England Biolabs) according to the manufacturer’s protocol. Briefly, 100 μg of fetuin was incubated in deglycosylation mix buffer 2 at 75°C for 10 min. After the mixture had cooled, protein deglycosylation mix II was added, with the exception of one tube to be used as a positive control for CpaA activity. The glycosylated and deglycosylated fetuin proteins were then used as substrates to test CpaA activity as described above.

### Glycan array.

The glycan array binding analysis was performed at the Consortium of Functional Glycomics (CFG). Two concentrations of purified CpaA (cfg_rRequest_3530) and CpaA_E520A_ (cfg_rRequest_3617) were screened with version 5.4 of the CFG printed array, which consists of 585 glycans in replicates of 6, according to the standard procedure of the CFG.

### Flow cytometry.

HeLa cells were maintained in Dulbecco’s minimal essential medium (DMEM) with high glucose supplemented with 10% (vol/vol) heat-inactivated fetal bovine serum (FBS) (Gibco) at 37°C and 5% CO_2_. HEK-293 cells were maintained in Eagle’s minimal essential medium (EMEM) with 10% (vol/vol) heat-inactivated FBS (Gibco) at 37°C and 5% CO_2_. Cells were seeded in 60-mm plates at a density of 1 × 10^6^ cells per plate a day prior to each experiment. For infection assays, freshly transformed A. nosocomialis M2 cells with plasmids pWH-*cpaA*-*his*-*cpaB* and pMFH32 were incubated overnight in LB supplemented with tetracycline. Stationary-phase cultures were normalized to an OD of 1 and washed three times with phosphate-buffered saline (PBS) before addition to cells at the indicated multiplicity of infection (MOI) in the respective cell medium. Infected cells were incubated for 2.5 h at 37°C and 5% CO_2_. Alternatively, cells were treated with 2 or 5 μg/ml of purified CpaA or CpaA_E520A_, as indicated, and incubated for 1 h at 37°C and 5% CO_2_. Following infection or treatment with purified protein, eukaryotic cells were washed with PBS (twice) and harvested using 0.05% trypsin (Corning). Cells were blocked with 2% (vol/vol) FBS in PBS and labeled with fluorophore-conjugated primary antibodies against CD55 (phycoerythrin [PE]; Sino Biological), and CD46 (allophycocyanin [APC]; Invitrogen). All incubations were carried out for 45 min on ice, and cells were washed three times with PBS. Labeled cells were fixed with 4% (vol/vol) paraformaldehyde in PBS for 10 min, washed with PBS, resuspended in PBS to a cell concentration of 1 × 10^6^ cell/ml, and filtered prior to analysis. Flow cytometry was performed on a three-laser, 8-color FACSCanto II cytometer (BD Biosciences). Experiments were performed in technical triplicates, with 10,000 events analyzed per sample. Data were analyzed using FlowJo 10.6 (FlowJo LLC), and individual values from three independent experiments (each experiment has at least three technical replicates) were combined and analyzed by two-way analysis of variance (ANOVA) using GraphPad Prism.

### Tryptic digest of gel-separated protein bands.

CpaA digested proteins were separated using SDS-PAGE, fixed, and visualized with Coomassie blue or InstantBlue, as described above. Bands of interest were excised and processed as previously described (37). Briefly, gel bands were first destained in a solution of 50 mM NH_4_HCO_3_–50% ethanol for 20 min at room temperature with shaking at 750 rpm. Destained bands were dehydrated with 100% ethanol, vacuum dried for 20 min, and then rehydrated in 50 mM NH_4_HCO_3_ plus 10 mM dithiothreitol (DTT). Protein bands were reduced for 60 min at 56°C with shaking then washed twice in 100% ethanol for 10 min to remove residual DTT. Reduced ethanol washed samples were sequentially alkylated with 55 mM iodoacetamide in 50 mM NH_4_HCO_3_ in the dark for 45 min at room temperature. Alkylated samples were then washed with 50 mM NH_4_HCO_3_ followed by 100% ethanol twice for 5 min to remove residual iodoacetamide and then vacuum dried. Alkylated samples were then rehydrated with 12 ng/μl trypsin (Promega) in 40 mM NH_4_HCO_3_ at 4°C for 1 h. Excess trypsin was removed, and gel pieces were covered in 40 mM NH_4_HCO_3_ and incubated overnight at 37°C. Peptides were concentrated and desalted using C_18_ stage tips (57, 58) before analysis by liquid chromatography (LC)-MS.

### Identification of CpaA digestion products and cleavage sites using reverse-phase LC-MS.

Purified peptides prepared were resuspend in buffer A* (0.1% trifluoroacetic acid [TFA], 2% acetonitrile) and separated using a two-column chromatography setup composed of a PepMap100 C_18_ 20-mm by 75-μm trap and a PepMap C_18_ 500-mm by 75-μm analytical column (Thermo Fisher Scientific). Samples were concentrated on the trap column at 5 μl/min for 5 min with buffer A (0.1% formic acid, 2% dimethyl sulfoxide [DMSO]) and infused into an Orbitrap Fusion Lumos Tribrid mass spectrometer (Thermo Fisher Scientific) at 300 nl/min via the analytical column using a Dionex Ultimate 3000 ultraperformance liquid chromatograph (UPLC) (Thermo Fisher Scientific). Gradients (45 or 65 min) were run for each sample, altering the buffer composition from 1% buffer B (0.1% formic acid, 77.9% acetonitrile, 2% DMSO) to 28% B over 20 or 40 min, then from 28% B to 40% B over 5 min, and then from 40% B to 100% B over 2 min; the composition was held at 100% B for 3 min, dropped to 3% B over 5 min, and held at 3% B for another 10 min. For 45-min gradients, the Lumos mass spectrometer was operated in a data-dependent mode automatically switching between the acquisition of a single Orbitrap MS scan (240,000 resolution) every 3 s and MS2 events. For each ion selected, dissociation parameters were as follows. For collision-induced dissociation (CID), Fourier transform MS (FTMS) was at 15,000 resolution, maximum fill time was 100 ms, and automatic gain control (AGC) was 2 × 10^5^. For higher-energy collisional dissociation (HCD), FTMS was at 15,000 resolution, maximum fill time was 120 ms, normalized collision energy was 35, and AGC was 2 × 10^5^. For electron transfer–higher-energy collision dissociation (EThcD), FTMS was at 15,000 resolution, maximum fill time was 120 ms, supplementary activation was 15%, and AGC was 2 × 10^5^. For 65-min gradients, the Lumos mass spectrometer was operated in a data-dependent mode, automatically switching between the acquisition of a single Orbitrap MS scan (120,000 resolution) every 3 s and MS2 HCD scans of precursors (normalized collision energy [NCE], 30%; maximal injection time of 22 ms; AGC, 5 × 10^4^ with a resolution of 15,000). HexNAc oxonium ion (204.087 *m/z*) product-dependent MS/MS analysis (59) was used to trigger two additional scans of potential glycopeptides, a CID scan (ion trap MS [ITMS]; maximum fill time, 100 ms, AGC, 5 × 10^4^) and an EThcD scan (FTMS, 30,000 resolution; maximum fill time, 200 ms; supplementary activation, 15%; AGC, 2 × 10^5^) scan.

### Mass spectrometry data analysis.

The assessment of the protein coverage within CpaA-digested bands and the identification of mucin glycopeptides was accomplished using MaxQuant (v1.5.3.30) (60). Searches were performed against the custom databases populated with the protein sequence of the recombinant proteins of interest with carbamidomethylation of cysteine set as a fixed modification. Searches were performed with semitrypsin cleavage specificity allowing 2 miscleavage events. For the identification of glycopeptide, multiple searches were performed on each sample, allowing oxidation of methionine and a maximum of three glycan variable modifications using (i) HexNAc (S/T), HexHexNAc (S/T), HexNAcHexNAc (S/T) or (ii) HexNAc (S/T), Hex(1)HexNAc(1)NeuAc(1) (S/T), Hex(2)HexNAc(1)NeuAc(1) (S/T). The precursor mass tolerance was set to 20 ppm for the first search and 10 ppm for the main search, with a maximum false discovery rate (FDR) of 1.0% set for protein and peptide identifications. The resulting protein group output was processed within the Perseus (v1.4.0.6) (60) analysis environment to remove reverse matches and common protein contaminates. Semitryptic peptides and semitryptic glycopeptides were manually inspected for correctness. Annotation of MS-MS provided within the supplementary data was undertaken using the Interactive Peptide Spectral Annotator (60).

### Molecular modeling.

All computational work was performed using the 2019 Molecular Operating Environment (MOE 2019.01) software suite (Chemical Computing Group ULC, Montreal, Canada) (61). The X-ray crystal structures of the metzincin enzymes CpaA (6O38) (13) and serralysin (3VI1), as well as the gluzincin enzymes IMPa (5KDX, (16), ZmpB (5KDU and 5KDS) (16), and BT4244 (5KD8) (16), were overlaid using the residues of the highly conserved alpha helix within their active sites (Table S2).

10.1128/mBio.02033-20.2 TABLE S2 Residues of the active site alpha helix used to overlay enzyme crystal structures. Conserved histidine residues are noted in bold. Download Table S2, PDF file, 0.1 MB. Copyright © 2020 Haurat et al. 2020 Haurat et al. This content is distributed under the terms of the Creative Commons Attribution 4.0 International license.

Following a previously described protocol (22), the crystal structure of CpaA was prepared by adding unresolved side chains and hydrogens as well as capping termini with acetyl or *N*-methyl (*N*-Me) groups. Notably, the crystallographic chaperone protein CpaB was not included during this study. This protein arranges its C-terminal tail into the CpaA catalytic site, similar to zymogens of related metallopeptidases (62), and we assume that this portion of CpaB is displaced by substrate.

Peptidomimetic and peptidic ligands have been observed to bind in a similar conformation within the metzincin active site (44, 45), using the P2-P1′ ligand residues to form an antiparallel β-sheet with a β-strand of the enzyme active site. Similar to previous work (22), the crystallographic peptide ligand bound to serralysin (RPKPQQ) was used as a scaffold to construct the peptide portion (Ac–EAPSA–*N*-Me) of the fetuin substrate fragment bound to the CpaA model (Table S2). Related gluzincin enzymes were found to have glycan-containing ligands near the P1′ site of their enzyme active sites (16). Thus, the crystallographic glycan bound to IMPa (Galβ1-3GalNAcα1) (5KDX) (16) and ZmpB (Galβ1-3(Neu5Acα2-6)GalNAcα1) (5KDU) (16) were grafted onto the fetuin peptide fragment to generate corresponding glycoforms, and the Glycan Fragment Database (63) was used to identify a glycan (Neu5Acα2-3Galβ1-3GalNAcα1) (2CWG) (64) to generate the final glycoform (Table S2).

The glycopeptides were docked into the CpaA model in three steps: conformational search, virtual screen, and minimization. This process allowed exploration of all reasonable conformations of each glycopeptide prior to induced-fit docking with the CpaA model. Each glycopeptide underwent a conformational search using the Amber10:EHT force field (65) to generate a corresponding library of conformers. During this step, the side chains of the peptide and the pendant groups of each sugar were allowed to freely rotate; any sialic acid moieties were also allowed to move freely. All other atoms were fixed. This process generated small libraries of approximately 4,000 conformers for each glycopeptide species. The conformers of each glycopeptide library underwent virtual screening with the CpaA model, again using the Amber10:EHT force field. During this process, all atoms of the enzyme and each glycopeptide were fixed, and the docking score for each resulting complex was calculated using the GBVI/WSA dG scoring function (66). Finally, the top 10 complexes identified from the virtual screening were subsequently minimized using the Amber10:EHT force field. The glycopeptide substrate, the catalytic zinc ion, and residues of the CpaA model having atoms within 10 Å of the substrate were allowed to move; all other residues were fixed, and solvent molecules were omitted. The best, most consistent complexes are shown.

This screening and minimization process was repeated for the Galβ1-3GalNAcα1-modified glycopeptide conformer library with the mutant CpaA models CpaAW493F, CpaAW493L, and CpaAW493A. In all cases, the dihedral angles of the peptide backbones (ϕ, ψ) and side chains (χ) as well as the initial glycosidic linkages (ϕ, ψ) of the docked substrates were measured to ensure proper geometry.

### Data availability.

The mass spectrometry proteomics data have been deposited in the ProteomeXchange Consortium database via the PRIDE (67) partner repository with the data set identifier PXD019941.
