# Supplementary figures and images for: The Glycoprotease CpaA Secreted by Medically Relevant Acinetobacter Species Targets Multiple O-Linked Host Glycoproteins
Source: mBio. 2020 Oct 6;11(5):e02033-20. doi: 10.1128/mBio.02033-20 (PMC7542363; doi:10.1128/mBio.02033-20)

**A**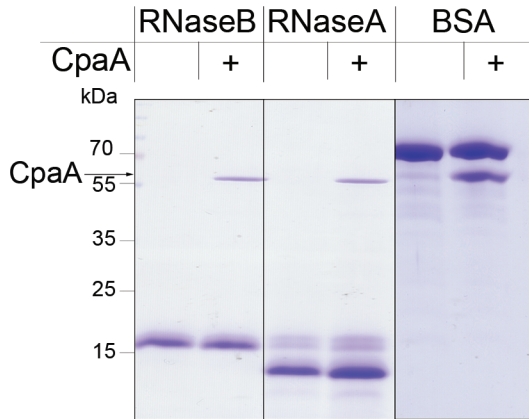**B**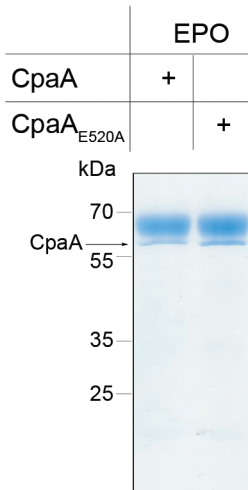

Supplement: FIG S1 [file mBio.02033-20-sf001.pdf]

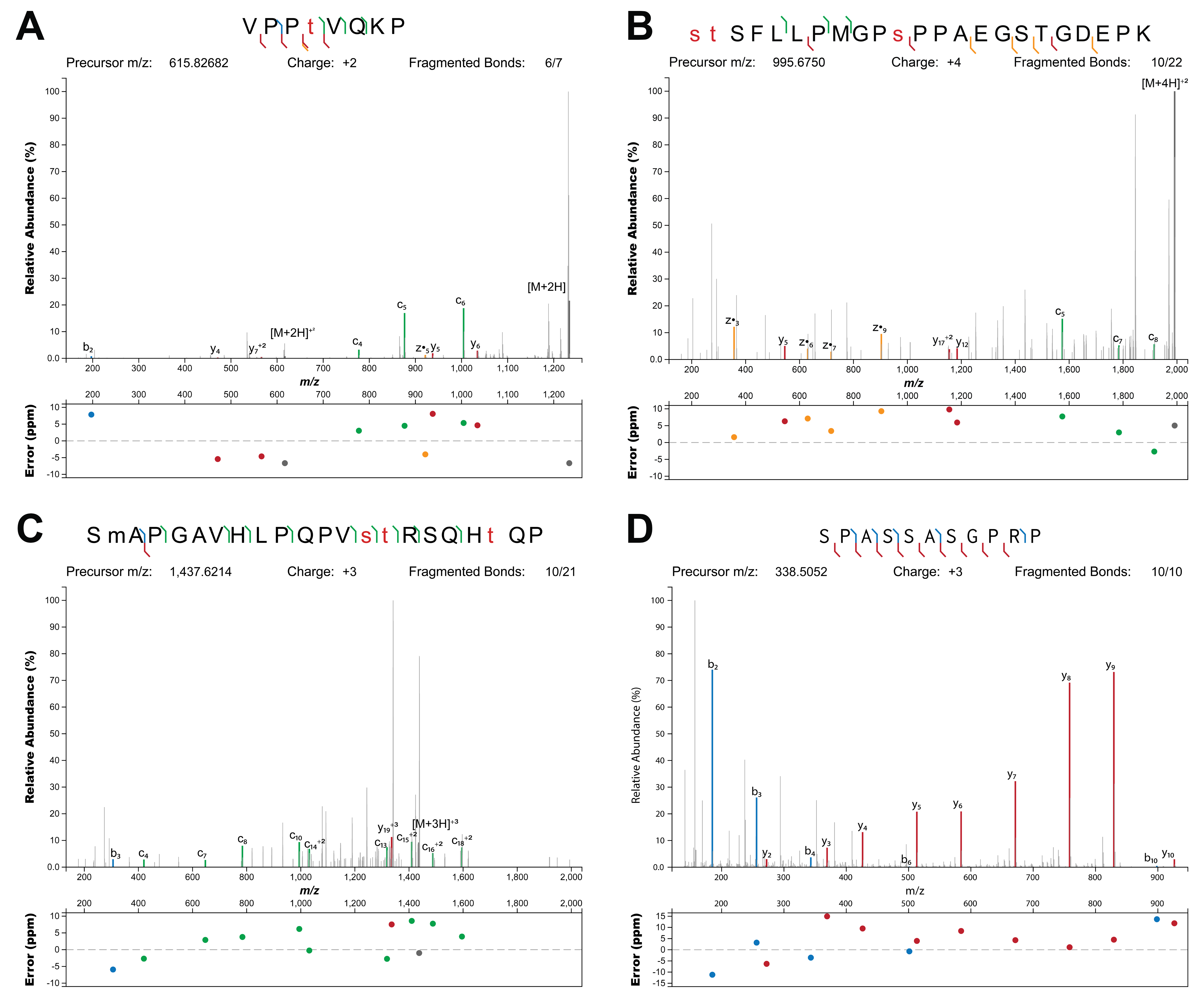

Supplement: FIG S2 [file mBio.02033-20-sf002.tif]

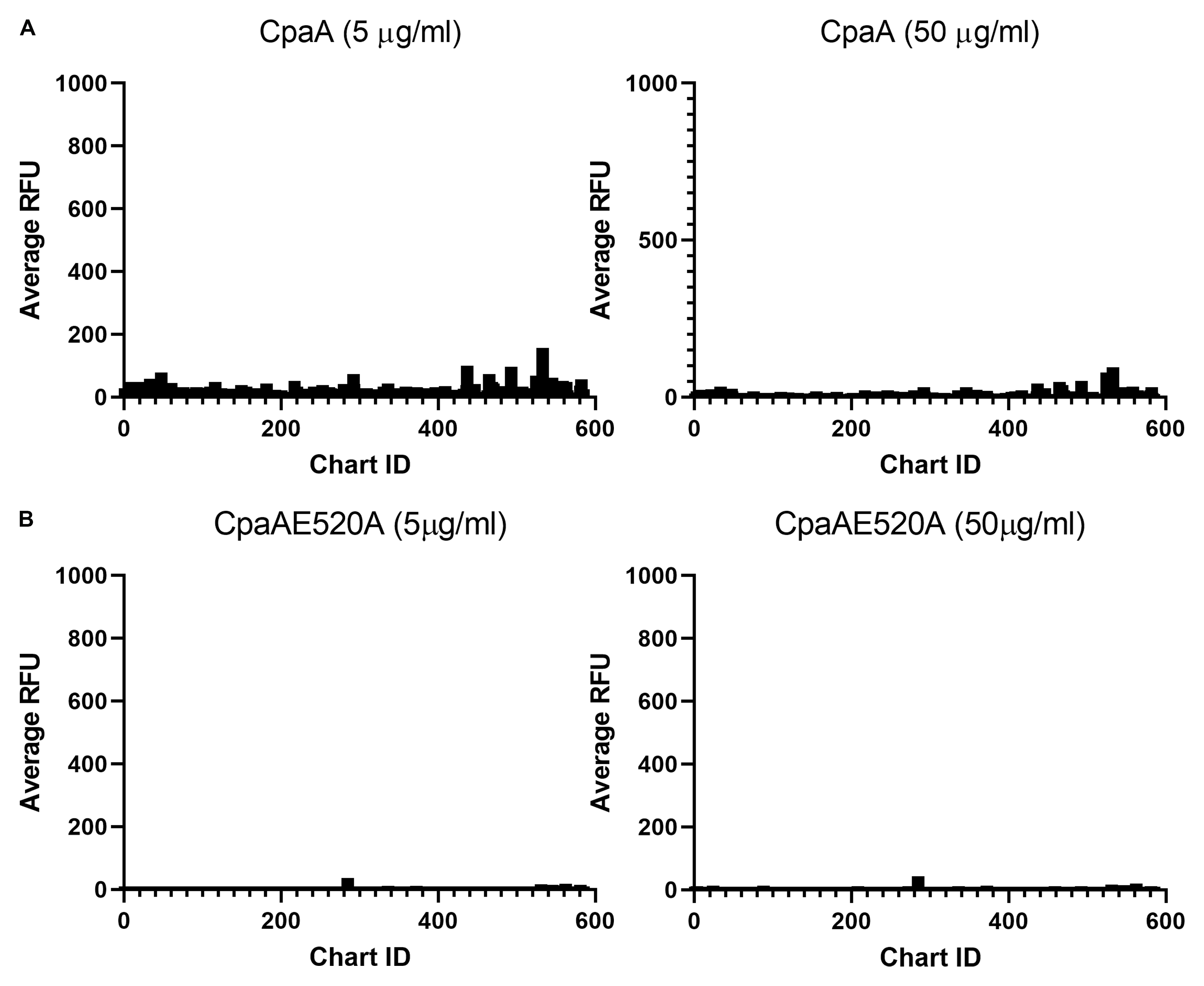

Supplement: FIG S3 [file mBio.02033-20-sf003.tif]

**A**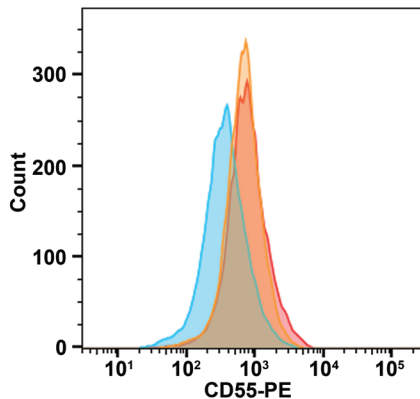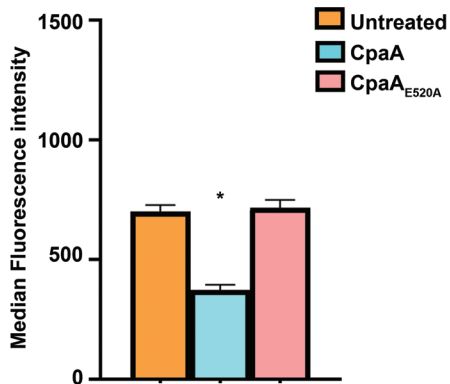**B**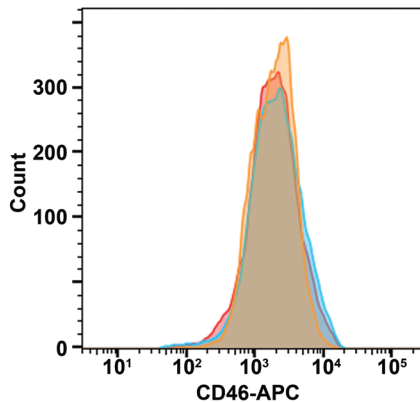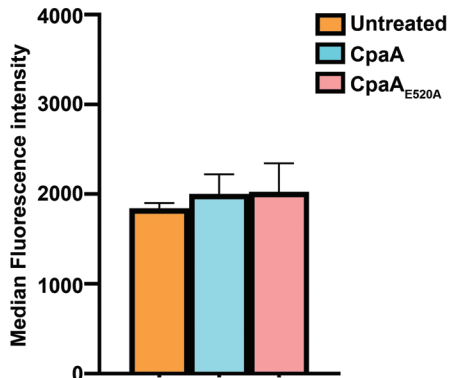

Supplement: FIG S4 [file mBio.02033-20-sf004.pdf]

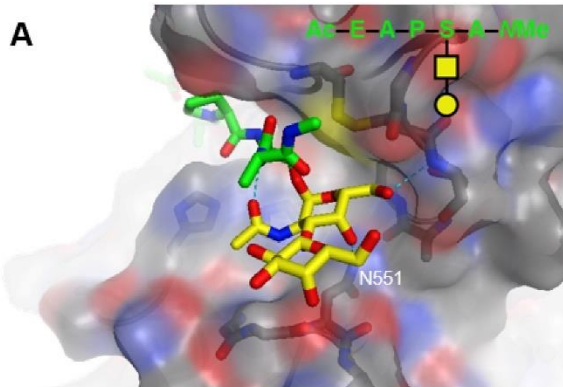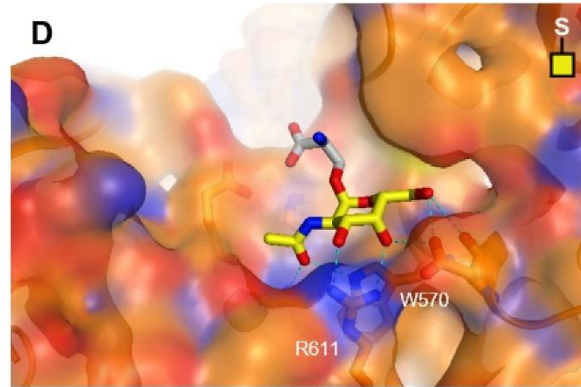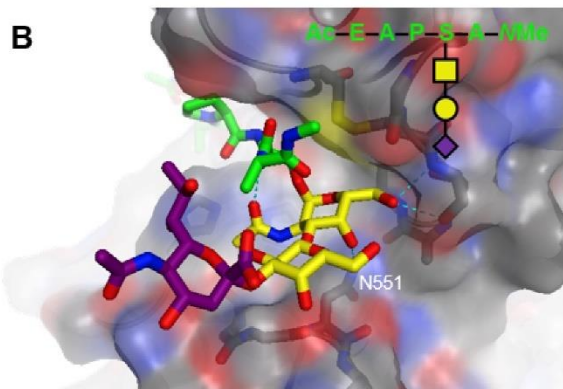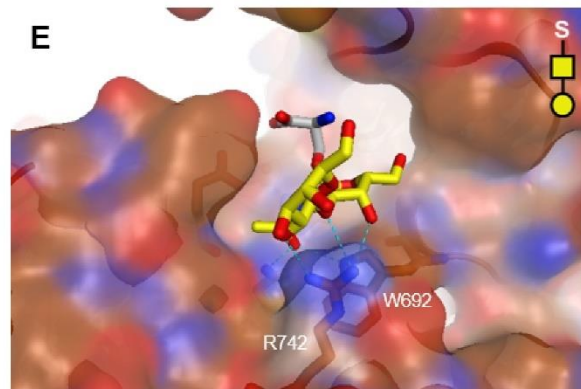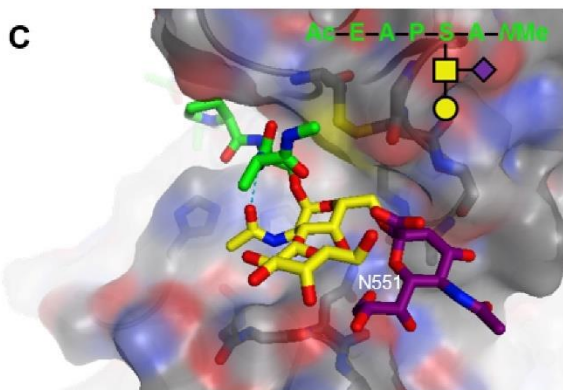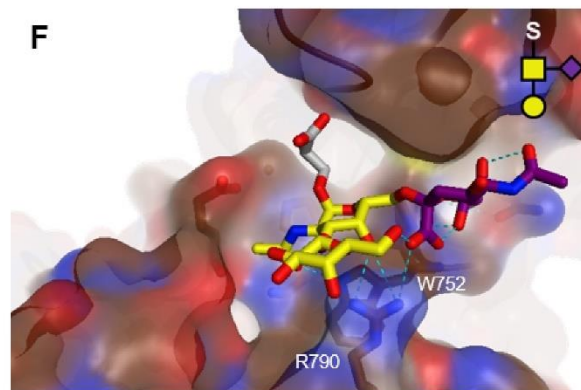

■ GalNAc

● Gal

◆ Neu5Ac

Supplement: FIG S5 [file mBio.02033-20-sf005.pdf]

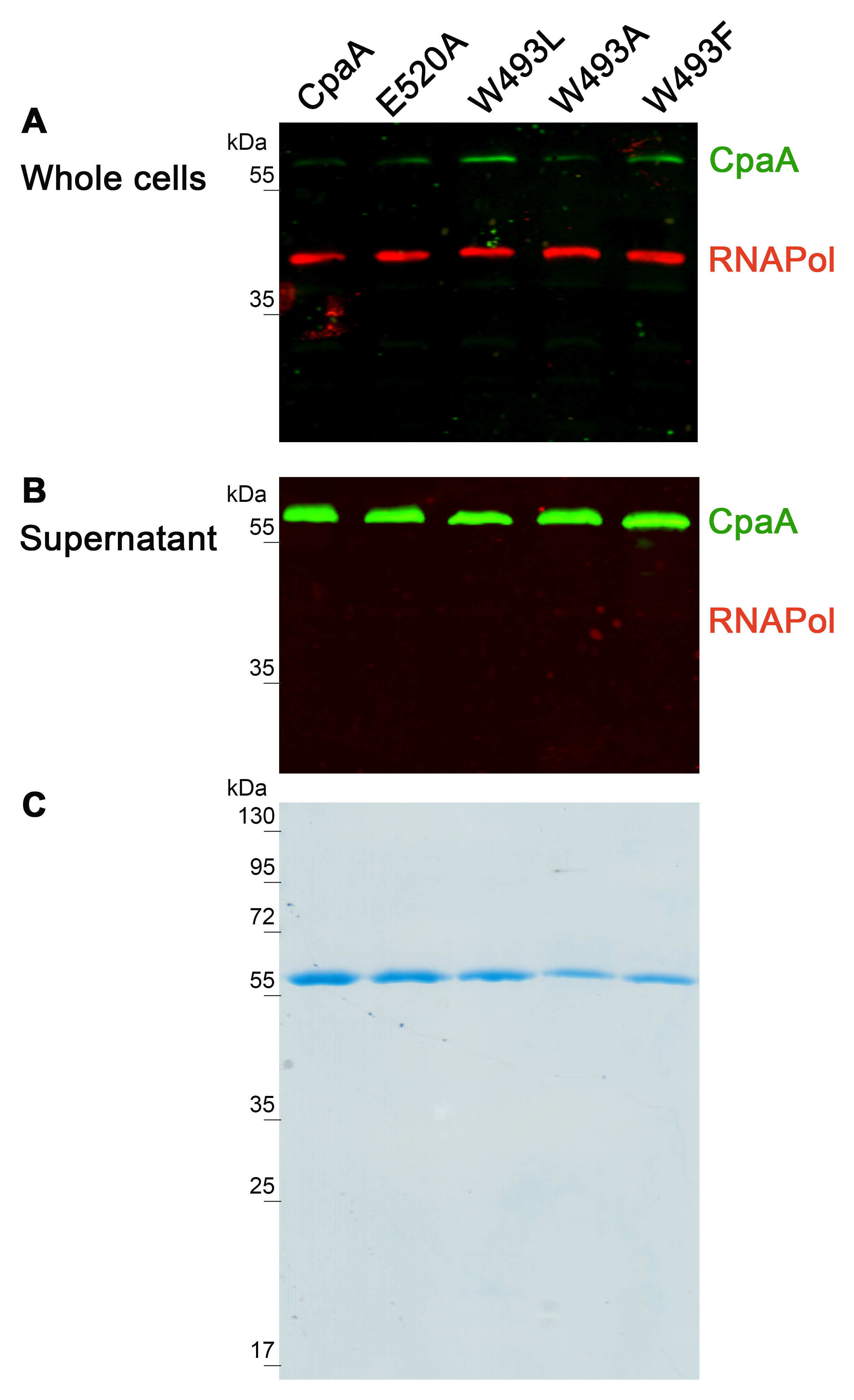

Supplement: FIG S6 [file mBio.02033-20-sf006.tif]

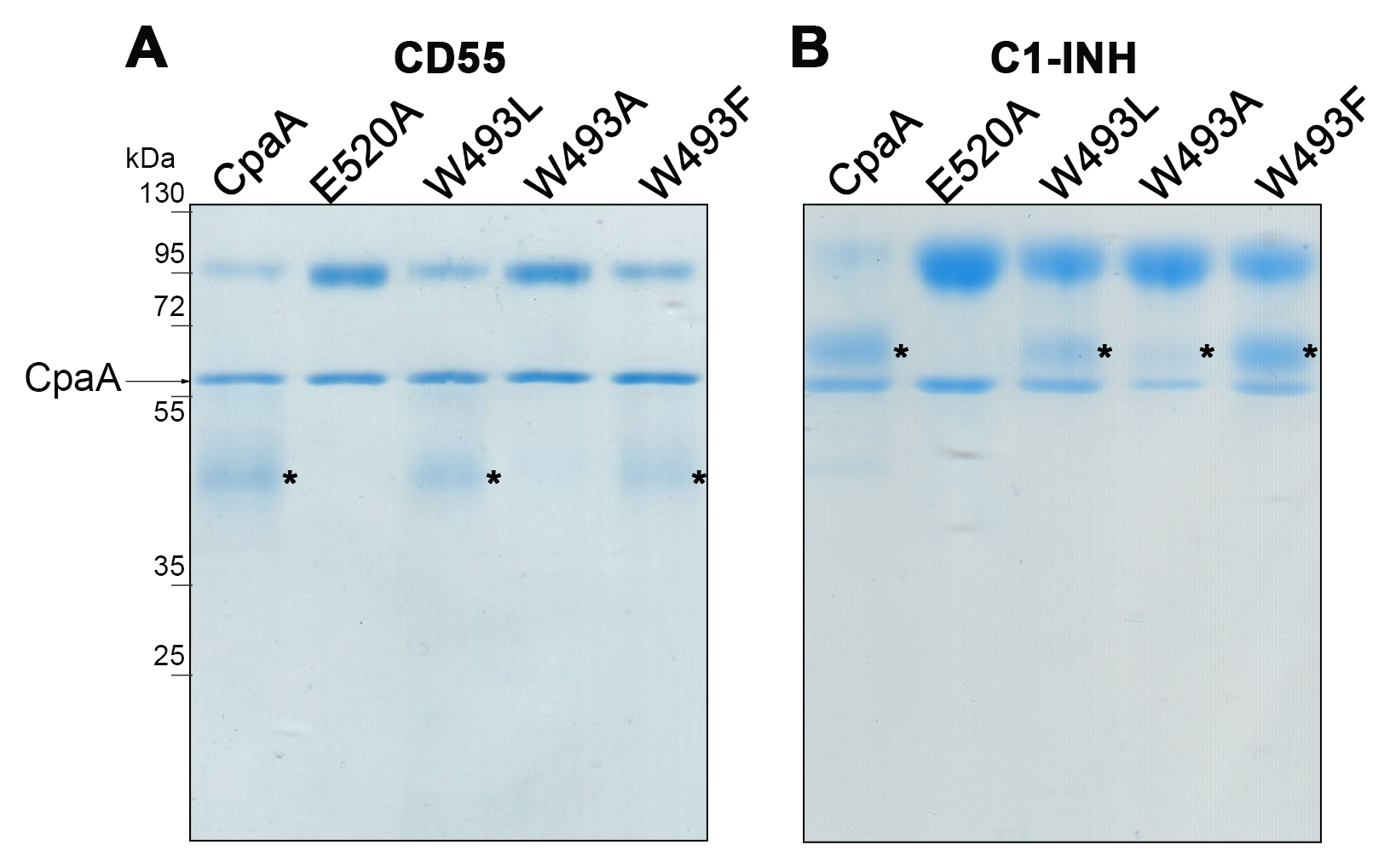

Supplement: FIG S7 [file mBio.02033-20-sf007.tif]
