# Supplementary material for: The Glycoprotease CpaA Secreted by Medically Relevant Acinetobacter Species Targets Multiple O-Linked Host Glycoproteins
Source: mBio. 2020 Oct 6;11(5):e02033-20. doi: 10.1128/mBio.02033-20 (PMC7542363; doi:10.1128/mBio.02033-20)
Supplement: FIG S8 [file mBio.02033-20-sf008.pdf]

```
AN_M2        1 VNFKLKTSLIIGAIVASSLVYAATVLSPNQNNNSGSIPTGYSDLEFSLANGNWVKNLSLP
AB031        1 MNFKLKTSLIIGAIVASSLVYAATVLSPNQNNNSGSIPSGYSDLEFNLANGNWVKNLTLP
UPAB1        1 MNFKLKTSLIIGAIVASSLVYAATVLSPNQNNNSGSIPSGYSDLEFNLANGNWVKNLTLP
J15          1 MNFKLKTSLIIGAIVASSLVYAATVLSPNQNNNSGSIPSGYSDLEFNLANGNWVKNLTLP
MRSN21681    1 MNFKLKTSLIIGAIVASSLVYAATVLSPNQNNNSGSIPSGYSDLEFNLANGNWVKNLTLP
MRSN11669    1 MNFKLKTSLIIGAIVASSLVYAATVLSPNQNNNSGSIPSGYSDLEFNLANGNWVKNLTLP
MRSN14237    1 MNFKLKTSLIIGAIVASSLVYAATVLSPNQNNNSGSIPSGYSDLEFNLANGNWVKNLTLP
Ab04         1 MNFKLKTSLIIGAIVASSLVYAATVLSPNQNNNSGSIPSGYSDLEFNLANGNWVKNLTLP
XH858        1 MNFKLKTSLIIGAIVASSLVYAATVLSPNQNNNSGSIPSGYSDLEFNLANGNWVKNLTLP
NIPH_335     1 MNFKLKTSLIIGAIVASSLVYAATVLSPNQNNNSGSIPSGYSDLEFNLANGNWVKNLTLP
OIFC098      1 MNFKLKTSLIIGAIVASSLVYAATVLSPNQNNNSGSIPSGYSDLEFNLANGNWVKNLTLP
1419130      1 MNFKLKTSLIIGAIVASSLVYAATVLSPNQNNNSGSIPSGYSDLEFNLANGNWVKNLTLP
NIPH_601     1 MNFKLKTSLIIGAIVASSLVYAATVLSPNQNNNSGSIPSGYSDLEFNLANGNWVKNLTLP
consensus    1 mNFKLKTSLIIGAIVASSLVYAATVLSPNQNNNSGSIPsGYSDLEFnLANGNWVKNLtLP

AN_M2       61 TNANNSDKITIRSSAAYSSYLDTSNTNIPLEVLKINSGDVYQFIFNSSQNKWIAQLATVS
AB031       61 TSANNLDKITIRSSAAYSSYLDTSNTNIPLEVLKINSGDVYQFIFNSSQNKWIAQLATVS
UPAB1       61 TSANNLDKITIRSSAAYSSYLDTSNTNIPLEVLKINSGDVYQFIFNSSQNKWIAQLATVS
J15         61 ISANNLDKITIRSSAAYSSYLDTSNTNIPLEVLKINSGDVYQFIFNSSQNKWIAQLATVS
MRSN21681   61 TSANNLDKITIRSSAAYSSYLDTSNTNIPLEVLKINSGDVYQFIFNSSQNKWIAQLATVS
MRSN11669   61 TSANNLDKITIRSSAAYSSYLDTSNTNIPLEVLKINSGDVYQFIFNSSQNKWIAQLATVS
MRSN14237   61 TSANNLDKITIRSSAAYSSYLDTSNTNIPLEVLKINSGDVYQFIFNSSQNKWIAQLATVS
Ab04        61 ISANNLDKITIRSSAAYSSYLDTSNTNIPLEVLKINSGDVYQFIFNSSQNKWIAQLATVS
XH858       61 ISANNLDKITIRSSAAYSSYLDTSNTNIPLEVLKINSGDVYQFIFNSSQNKWIAQLATVS
NIPH_335    61 ISANNLDKITIRSSAAYSSYLDTSNTNIPLEVLKINSGDVYQFIFNSSQNKWIAQLATVS
OIFC098     61 ISANNLDKITIRSSAAYSSYLDTSNTNIPLEVLKINSGDVYQFIFNSSQNKWIAQLATVS
1419130     61 ISANNLDKITIRSSAAYSSYLDTSNTNIPLEVLKINSGDVYQFIFNSSQNKWIAQLATVS
NIPH_601    61 ISANNLDKITIRSSAAYSSYLDTSNTNIPLEVLKINSGDVYQFIFNSSQNKWIAQLATVS
consensus   61 isANNlDKITIRSSAAYSSYLDTSNTNIPLEVLKINSGDVYQFIFNSSQNKWIAQLATVS

AN_M2      121 PTTGSNYELIPLTTATMQKVLIQDDKWAQTIALPSDVRDGTTVQVVSTASVSSDIDKTNL
AB031      121 PTNGATYEVVPLTTASMQKVLIQNEKWAQTIALPSDVRDGTTVQVVSTASASSDIDKTNL
UPAB1      121 PTNGATYEVVPLTTASMQKVIIQNDKWAQTIALPSDVRDGTTVQVVSTASTSSEIDKTNL
J15        121 PTNGATYEVVPLTTASMQKVIIQNDKWAQTIALPSDVRDGTTVQVVSTASTSSDIDKTNL
MRSN21681  121 PTNGATYEVVPLTTASMQKVLIQNEKWAQTIALPSDVRDGTTVQVVSTASASSDIDKTNL
MRSN11669  121 PTNGATYEVVPLTTASMQKVLIQNEKWAQTIALPSDVRDGTTVQVVSTASASSDIDKTNL
MRSN14237  121 PTNGATYEVVPLTTASMQKVIIQNDKWAQTIALPSDVRDGTTVQVVSTASTSSEIDKTNL
Ab04       121 PTNGATYEVVPLTTASMQKVIIQNDKWAQTIALPSDVRDGTTVQVVSTASTSSDIDKTNL
XH858      121 PTNGATYEVVPLTTASMQKVIIQNDKWAQTIALPSDVRDGTTVQVVSTASTSSDIDKTNL
NIPH_335   121 PTNGATYEVVPLTTASMQKVIIQNDKWAQTIALPSDVRDGTTVQVVSTASTSSDIDKTNL
OIFC098    121 PTNGATYEVVPLTTASMQKVIIQNDKWAQTIALPSDVRDGTTVQVVSTASTSSDIDKTNL
1419130    121 PTNGATYEVVPLTTASMQKVIIQNDKWAQTIALPSDVRDGTTVQVVSTASTSSDIDKTNL
NIPH_601   121 PTNGATYEVVPLTTASMQKVIIQNDKWAQTIALPSDVRDGTTVQVVSTASTSSDIDKTNL
consensus  121 PTnGatYEvvPLTTAsMQKViIQndKWAQTIALPSDVRDGTTVQVVSTAStSSdIDKTNL

AN_M2      181 LFPSSFTLKNGSEYWFKYYSALGKWVPEYIKPQKLNVQQIGTSLAAVNSPLTEIAFGDGN
```

```
AB031       181 LFPSSFILKNGSEYWFKYYSALGKWVPEYIKPQKLNVQQIGTSLATVNSPLTEVSFGDGN
UPAB1       181 LFPSSFILKNGSEYWFKYYSALGKWVPEYIKPQKLNVQQIGTSLATVNSPLTEIAFGDGN
J15         181 LFPSSFSLKNGSEYWFKYYSALGKWVPEYIKPQKLNVQQIGTSLAAVNSPLTEVSFGDGN
MRSN21681   181 LFPSSFILKNGSEYWFKYYSALGKWVPEYIKPQKLNVQQIGTSLATVNSPLTEVSFGDGN
MRSN11669   181 LFPSSFILKNGSEYWFKYYSALGKWVPEYIKPQKLNVQQIGTSLATVNSPLTEVSFGDGN
MRSN14237   181 LFPSSFILKNGSEYWFKYYSALGKWVPEYIKPQKLNVQQIGTSLATVNSPLTEIAFGDGN
Ab04        181 LFPSSFSLKNGSEYWFKYYSALGKWVPEYIKPQKLNVQQIGTSLAAVNSPLTEVSFGDGN
XH858       181 LFPSSFSLKNGSEYWFKYYSALGKWVPEYIKPQKLNVQQIGTSLAAVNSPLTEVSFGDGN
NIPH_335    181 LFPSSFSLKNGSEYWFKYYSALGKWVPEYIKPQKLNVQQIGTSLAAVNSPLTEVSFGDGN
OIFC098     181 LFPSSFSLKNGSEYWFKYYSALGKWVPEYIKPQKLNVQQIGTSLAAVNSPLTEVSFGDGN
1419130     181 LFPSSFSLKNGSEYWFKYYSALGKWVPEYIKPQKLNVQQIGTSLAAVNSPLTEVSFGDGN
NIPH_601    181 LFPSSFSLKNGSEYWFKYYSALGKWVPEYIKPQKLNVQQIGTSLAAVNSPLTEVSFGDGN
consensus   181 LFPSSFsLKNGSEYWFKYYSALGKWVPEYIKPQKLNVQQIGTSLAaVNSPLTEvsFGDGN

AN_M2       241 WVSNFTLPTTANDRDRIIIKSTATWSAKINNTNVNSQATLTLKTGDQYEFMYVSDKGYWQ
AB031       241 WVSNFTLPTTASDRDKIIIKSTATWSAKINNTNINSQATLTLKTGDQYEFMYVSDKGYWQ
UPAB1       241 WVSNFTLPTTASDRDRIIIKSTATWSAKINNTNINSQATLTLKTGDQYEFMYVSDKGYWQ
J15         241 WVSNFTLPTTASDRDRIIIKSTATWSAKINNTNINSQATLTLKTGDQYEFMYVSDKGYWQ
MRSN21681   241 WVSNFTLPTTASDRDKIIIKSTATWSAKINNTNINSQATLTLKTGDQYEFMYVSDKGYWQ
MRSN11669   241 WVSNFTLPTTASDRDKIIIKSTATWSAKINNTNINSQATLTLKTGDQYEFMYVSDKGYWQ
MRSN14237   241 WVSNFTLPTTASDRDRIIIKSTATWSAKINNTNINSQATLTLKTGDQYEFMYVSDKGYWQ
Ab04        241 WVSNFTLPTTASDRDRIIIKSTATWSAKINNTNINSQATLTLKTGDQYEFMYVSDKGYWQ
XH858       241 WVSNFTLPTTASDRDRIIIKSTATWSAKINNTNINSQATLTLKTGDQYEFMYVSDKGYWQ
NIPH_335    241 WVSNFTLPTTASDRDRIIIKSTATWSAKINNTNINSQATLTLKTGDQYEFMYVSDKGYWQ
OIFC098     241 WVSNFTLPTTASDRDRIIIKSTATWSAKINNTNINSQATLTLKTGDQYEFMYVSDKGYWQ
1419130     241 WVSNFTLPTTASDRDRIIIKSTATWSAKINNTNINSQATLTLKTGDQYEFMYVSDKGYWQ
NIPH_601    241 WVSNFTLPTTASDRDRIIIKSTATWSAKINNTNINSQATLTLKTGDQYEFMYVSDKGYWQ
consensus   241 WVSNFTLPTTAsDRDrIIIKSTATWSAKINNTNiNSQATLTLKTGDQYEFMYVSDKGYWQ

AN_M2       301 LISSPTKVIDSTATIPAILPNMTQPTLKVKLSTSNWQPTLQLPAQAQVGDKVVIVSNASA
AB031       301 LISSPTKVIDSAATIPATLPNMTQPTLKVKLSTSNWQPTLQLPAKAQVGDKVVIVSNASA
UPAB1       301 LISSPTKVIDSTATIPATLPNMTQPTLKVKLSTSNWQPTLQLPAKAQVGDKVVIVSNASA
J15         301 LISSPTKVIDSTATIPATLPNMTQPTLKVKLSTSNWQPTLQLPAKAQVGDKVVIVSNASA
MRSN21681   301 LISSPTKVIDSAATIPATLPNMTQPTLKVKLSTSNWQPTLQLPAKAQVGDKVVIVSNASA
MRSN11669   301 LISSPTKVIDSAATIPATLPNMTQPTLKVKLSTSNWQPTLQLPAKAQVGDKVVIVSNASA
MRSN14237   301 LISSPTKVIDSTATIPATLPNMTQPTLKVKLSTSNWQPTLQLPAKAQVGDKVVIVSNASA
Ab04        301 LISSPTKVIDSTATIPAILPNMTQPTLKVKLSTSNWQPTLQLPAQAQVGDKVVIVSNASA
XH858       301 LISSPTKVIDSTATIPAILPNMTQPTLKVKLSTSNWQPTLQLPAQAQVGDKVVIVSNASA
NIPH_335    301 LISSPTKVIDSTATIPAILPNMTQPTLKVKLSTSNWQPTLQLPAQAQVGDKVVIVSNASA
AN_M2         1 VNFKLKTSLIIGAIVASSLVYAATVLSPNQNNNSGSIPTGYSDLEFSLANGNWVKNLSLP
AB031         1 MNFKLKTSLIIGAIVASSLVYAATVLSPNQNNNSGSIPSGYSDLEFNLANGNWVKNLTLP
UPAB1         1 MNFKLKTSLIIGAIVASSLVYAATVLSPNQNNNSGSIPSGYSDLEFNLANGNWVKNLTLP
J15           1 MNFKLKTSLIIGAIVASSLVYAATVLSPNQNNNSGSIPSGYSDLEFNLANGNWVKNLTLP
MRSN21681     1 MNFKLKTSLIIGAIVASSLVYAATVLSPNQNNNSGSIPSGYSDLEFNLANGNWVKNLTLP
MRSN11669     1 MNFKLKTSLIIGAIVASSLVYAATVLSPNQNNNSGSIPSGYSDLEFNLANGNWVKNLTLP
MRSN14237     1 MNFKLKTSLIIGAIVASSLVYAATVLSPNQNNNSGSIPSGYSDLEFNLANGNWVKNLTLP
Ab04          1 MNFKLKTSLIIGAIVASSLVYAATVLSPNQNNNSGSIPSGYSDLEFNLANGNWVKNLTLP
```

```
XH858         1  MNFKLKTSLIIGAIVASSLVYAATVLSPNQNNNSGSIPSGYSDLEFNLANGNWVKNLTLP
NIPH_335      1  MNFKLKTSLIIGAIVASSLVYAATVLSPNQNNNSGSIPSGYSDLEFNLANGNWVKNLTLP
OIFC098       1  MNFKLKTSLIIGAIVASSLVYAATVLSPNQNNNSGSIPSGYSDLEFNLANGNWVKNLTLP
1419130       1  MNFKLKTSLIIGAIVASSLVYAATVLSPNQNNNSGSIPSGYSDLEFNLANGNWVKNLTLP
NIPH_601      1  MNFKLKTSLIIGAIVASSLVYAATVLSPNQNNNSGSIPSGYSDLEFNLANGNWVKNLTLP
consensus     1  mNFKLKTSLIIGAIVASSLVYAATVLSPNQNNNSGSIPsGYSDLEFnLANGNWVKNLtLP

AN_M2        61  TNANNSDKITIRSSAAYSSYLDTSNTNIPLEVLKINSGDVYQFIFNSSQNKWIAQLATVS
AB031        61  TSANNLDKITIRSSAAYSSYLDTSNTNIPLEVLKINSGDVYQFIFNSSQNKWIAQLATVS
UPAB1        61  TSANNLDKITIRSSAAYSSYLDTSNTNIPLEVLKINSGDVYQFIFNSSQNKWIAQLATVS
J15          61  ISANNLDKITIRSSAAYSSYLDTSNTNIPLEVLKINSGDVYQFIFNSSQNKWIAQLATVS
MRSN21681    61  TSANNLDKITIRSSAAYSSYLDTSNTNIPLEVLKINSGDVYQFIFNSSQNKWIAQLATVS
MRSN11669    61  TSANNLDKITIRSSAAYSSYLDTSNTNIPLEVLKINSGDVYQFIFNSSQNKWIAQLATVS
MRSN14237    61  TSANNLDKITIRSSAAYSSYLDTSNTNIPLEVLKINSGDVYQFIFNSSQNKWIAQLATVS
Ab04         61  ISANNLDKITIRSSAAYSSYLDTSNTNIPLEVLKINSGDVYQFIFNSSQNKWIAQLATVS
XH858        61  ISANNLDKITIRSSAAYSSYLDTSNTNIPLEVLKINSGDVYQFIFNSSQNKWIAQLATVS
NIPH_335     61  ISANNLDKITIRSSAAYSSYLDTSNTNIPLEVLKINSGDVYQFIFNSSQNKWIAQLATVS
OIFC098      61  ISANNLDKITIRSSAAYSSYLDTSNTNIPLEVLKINSGDVYQFIFNSSQNKWIAQLATVS
1419130      61  ISANNLDKITIRSSAAYSSYLDTSNTNIPLEVLKINSGDVYQFIFNSSQNKWIAQLATVS
NIPH_601     61  ISANNLDKITIRSSAAYSSYLDTSNTNIPLEVLKINSGDVYQFIFNSSQNKWIAQLATVS
consensus    61  isANNlDKITIRSSAAYSSYLDTSNTNIPLEVLKINSGDVYQFIFNSSQNKWIAQLATVS

AN_M2       121  PTTGSNYELIPLTTATMQKVLIQDDKWAQTIALPSDVRDGTTVQVVSTASVSSDIDKTNL
AB031       121  PTNGATYEVVPLTTASMQKVLIQNEKWAQTIALPSDVRDGTTVQVVSTASASSDIDKTNL
UPAB1       121  PTNGATYEVVPLTTASMQKVIIQNDKWAQTIALPSDVRDGTTVQVVSTASTSSEIDKTNL
J15         121  PTNGATYEVVPLTTASMQKVIIQNDKWAQTIALPSDVRDGTTVQVVSTASTSSDIDKTNL
MRSN21681   121  PTNGATYEVVPLTTASMQKVLIQNEKWAQTIALPSDVRDGTTVQVVSTASASSDIDKTNL
MRSN11669   121  PTNGATYEVVPLTTASMQKVLIQNEKWAQTIALPSDVRDGTTVQVVSTASASSDIDKTNL
MRSN14237   121  PTNGATYEVVPLTTASMQKVIIQNDKWAQTIALPSDVRDGTTVQVVSTASTSSEIDKTNL
Ab04        121  PTNGATYEVVPLTTASMQKVIIQNDKWAQTIALPSDVRDGTTVQVVSTASTSSDIDKTNL
XH858       121  PTNGATYEVVPLTTASMQKVIIQNDKWAQTIALPSDVRDGTTVQVVSTASTSSDIDKTNL
NIPH_335    121  PTNGATYEVVPLTTASMQKVIIQNDKWAQTIALPSDVRDGTTVQVVSTASTSSDIDKTNL
OIFC098     121  PTNGATYEVVPLTTASMQKVIIQNDKWAQTIALPSDVRDGTTVQVVSTASTSSDIDKTNL
1419130     121  PTNGATYEVVPLTTASMQKVIIQNDKWAQTIALPSDVRDGTTVQVVSTASTSSDIDKTNL
NIPH_601    121  PTNGATYEVVPLTTASMQKVIIQNDKWAQTIALPSDVRDGTTVQVVSTASTSSDIDKTNL
consensus   121  PTnGatYEvvPLTTAsMQKViIQndKWAQTIALPSDVRDGTTVQVVSTAStSSdIDKTNL

AN_M2       181  LFPSSFTLKNGSEYWFKYYSALGKWVPEYIKPQKLNVQQIGTSLAAVNSPLTEIAFGDGN
AB031       181  LFPSSFILKNGSEYWFKYYSALGKWVPEYIKPQKLNVQQIGTSLATVNSPLTEVSFGDGN
UPAB1       181  LFPSSFILKNGSEYWFKYYSALGKWVPEYIKPQKLNVQQIGTSLATVNSPLTEIAFGDGN
J15         181  LFPSSFSLKNGSEYWFKYYSALGKWVPEYIKPQKLNVQQIGTSLAAVNSPLTEVSFGDGN
MRSN21681   181  LFPSSFILKNGSEYWFKYYSALGKWVPEYIKPQKLNVQQIGTSLATVNSPLTEVSFGDGN
MRSN11669   181  LFPSSFILKNGSEYWFKYYSALGKWVPEYIKPQKLNVQQIGTSLATVNSPLTEVSFGDGN
MRSN14237   181  LFPSSFILKNGSEYWFKYYSALGKWVPEYIKPQKLNVQQIGTSLATVNSPLTEIAFGDGN
Ab04        181  LFPSSFSLKNGSEYWFKYYSALGKWVPEYIKPQKLNVQQIGTSLAAVNSPLTEVSFGDGN
XH858       181  LFPSSFSLKNGSEYWFKYYSALGKWVPEYIKPQKLNVQQIGTSLAAVNSPLTEVSFGDGN
```

```
NIPH_335   181 LFPSSFSLKNGSEYWFKYYSALGKWVPEYIKPQKLNVQQIGTSLAAVNSPLTEVSFGDGN
OIFC098    181 LFPSSFSLKNGSEYWFKYYSALGKWVPEYIKPQKLNVQQIGTSLAAVNSPLTEVSFGDGN
1419130    181 LFPSSFSLKNGSEYWFKYYSALGKWVPEYIKPQKLNVQQIGTSLAAVNSPLTEVSFGDGN
NIPH_601   181 LFPSSFSLKNGSEYWFKYYSALGKWVPEYIKPQKLNVQQIGTSLAAVNSPLTEVSFGDGN
consensus  181 LFPSSFsLKNGSEYWFKYYSALGKWVPEYIKPQKLNVQQIGTSLAaVNSPLTEvsFGDGN


1419130    301 LISSPTKVIDSTATIPATLPNMTQPTLKVKLSTSNWQPTLQLPAKAQVGDKVVIVSNASA
NIPH_601   301 LISSPTKVIDSTATIPATLPNMTQPTLKVKLSTSNWQPTLQLPAKAQVGDKVVIVSNASA
consensus  301 LISSPTKVIDStATIPAtLPNMTQPTLKVKLSTSNWQPTLQLPAkAQVGDKvVIVSNASA


AN_M2      361 DTYINAANGLSTAIKNGENRRFIYTAQGWTVDSYTIDMLLVSSPEVNSILGESAAKLRMI
AB031      361 DTYINAANGLSTAIKNGENRRFIYTAQGWTVDSYTIDMLLVSSPEVNSILGESAAKLRMI
UPAB1      361 DTYINAANGLSTAIKNGENRRFIYTAQGWTVDSYTIDMLLVSSPEVNSILGESAAKLRMI
J15        361 DTYINAANGLTTAIKNGENRRFIYTAQGWTVDSYTIDMLLVSSPEVNSILGDSAAKLRMI
MRSN21681  361 DTYINAANGLSTAIKNGENRRFIYTAQGWTVDSYTIDMLLVSSPEVNSILGESAAKLRMI
MRSN11669  361 DTYINAANGLSTAIKNGENRRFIYTAQGWTVDSYTIDMLLVSSPEVNSILGESAAKLRMI
MRSN14237  361 DTYINAANGLSTAIKNGENRRFIYTAQGWTVDSYTIDMLLVSSPEVNSILGESAAKLRMI
Ab04       361 DTYINAANGLSTAIKNGENRRFIYTAQGWTVDSYTIDMLLVSSPEVNSILGESAAKLRMI
XH858      361 DTYINAANGLSTAIKNGENRRFIYTAQGWTVDSYTIDMLLVSSPEVNSILGESAAKLRMI
NIPH_335   361 DTYINAANGLSTAIKNGENRRFIYTAQGWTVDSYTIDMLLVSSPEVNSILGESAAKLRMI
OIFC098    361 DTYINAANGLSTAIKNGENRRFIYTAQGWTVDSYTIDMLLVSSPEVNSILGESAAKLRMI
1419130    361 DTYINAANGLTTAIKNGENRRFIYTAQGWTVDSYTIDMLLVSSPEVNSILGDSAAKLRMI
NIPH_601   361 DTYINAANGLTTAIKNGENRRFIYTAQGWTVDSYTIDMLLVSSPEVNSILGDSAAKLRMI
consensus  361 DTYINAANGLsTAIKNGENRRFIYTAQGWTVDSYTIDMLLVSSPEVNSILGeSAAKLRMI


AN_M2      421 EGVNLTNLTAENSNARFYLRDVGYITYKIPAATLKEAISTGRDDTTVQNERKRILADGVY
AB031      421 EGVNLTNLTAENSNARFYLRNVGYLTYKIPAATLKEAISTGRDDTTVQNERKRVLADGVY
UPAB1      421 EGVNLTNLTAENSNARFYLRNVGYLTYKIPAATLKEAISTGRDDTTVQNERKRVLADGVY
J15        421 EGVNLTNLTAENSNARFYLRDVGYLTYKIPAATLKEAISTGRDDTTVQNERKRVLADGVY
MRSN21681  421 EGVNLTNLTAENSNARFYLRNVGYLTYKIPAATLKEAISTGRDDTTVQNERKRVLADGVY
MRSN11669  421 EGVNLTNLTAENSNARFYLRNVGYLTYKIPAATLKEAISTGRDDTTVQNERKRVLADGVY
MRSN14237  421 EGVNLTNLTAENSNARFYLRNVGYLTYKIPAATLKEAISTGRDDTTVQNERKRVLADGVY
Ab04       421 EGVNLTNLTAENSNARFYLRDVGYLTYKIPATTLKEAISTGRDDTTVQNERKRVLADGVY
XH858      421 EGVNLTNLTAENSNARFYLRDVGYLTYKIPATTLKEAISTGRDDTTVQNERKRVLADGVY
NIPH_335   421 EGVNLTNLTAENSNARFYLRDVGYLTYKIPATTLKEAISTGRDDTTVQNERKRVLADGVY
OIFC098    421 EGVNLTNLTAENSNARFYLRDVGYLTYKIPATTLKEAISTGRDDTTVQNERKRVLADGVY
1419130    421 EGVNLTNLTAENSNARFYLRDVGYLTYKIPAATLKEAISTGRDDTTVQNERKRVLADGVY
NIPH_601   421 EGVNLTNLTAENSNARFYLRDVGYLTYKIPAATLKEAISTGRDDTTVQNERKRVLADGVY
consensus  421 EGVNLTNLTAENSNARFYLRdVGYlTYKIPAaTLKEAISTGRDDTTVQNERKRvLADGVY
```

```
AN_M2      481 YQGNEPGDGGCGWAWINASAYNMIGANDIAGCSFAAMRHEVGHNLGLYHNGSTNIGSGFA
AB031      481 YQGNEPGDGGCGWAWINASAYNMIGANDIAGCSFAAMRHEVGHNLGLYHNGSTNIGSGFA
UPAB1      481 YQGNEPGDGGCGWAWINASAYNMIGANDIAGCSFAAMRHEVGHNLGLYHNGSTNIGSGFA
J15        481 YQGNEPGDGGCGWAWINASAYNMIGANDIAGCSFAAMRHEVGHNLGLYHNGSTNIGSGFA
MRSN21681  481 YQGNEPGDGGCGWAWINASAYNMIGANDIAGCSFAAMRHEVGHNLGLYHNGSTNIGSGFA
MRSN11669  481 YQGNEPGDGGCGWAWINASAYNMIGANDIAGCSFAAMRHEVGHNLGLYHNGSTNIGSGFA
MRSN14237  481 YQGNEPGDGGCGWAWINASAYNMIGANDIAGCSFAAMRHEVGHNLGLYHNGSTNIGSGFA
Ab04       481 YQGNEPGDGGCGWAWINASAYNMIGANDIAGCSFAAMRHEVGHNLGLYHNGSTNIGSGFA
XH858      481 YQGNEPGDGGCGWAWINASAYNMIGANDIAGCSFAAMRHEVGHNLGLYHNGSTNIGSGFA
NIPH_335   481 YQGNEPGDGGCGWAWINASAYNMIGANDIAGCSFAAMRHEVGHNLGLYHNGSTNIGSGFA
OIFC098    481 YQGNEPGDGGCGWAWINASAYNMIGANDIAGCSFAAMRHEVGHNLGLYHNGSTNIGSGFA
1419130    481 YQGNEPGDGGCGWAWINASAYNMIGANDIAGCSFAAMRHEVGHNLGLYHNGSTNIGSGFA
NIPH_601   481 YQGNEPSDGGCGWAWINASAYNMIGANDIAGCSFAAMRHEVGHNLGLYHNGSTNIGSGFA
consensus  481 YQGNEPgDGGCGWAWINASAYNMIGANDIAGCSFAAMRHEVGHNLGLYHNGSTNIGSGFA

AN_M2      541 HPLGSTAMGGNNINFYSSPYLYNPKYGVRLGEEGKIDAVSVINLNAQKISLYN
AB031      541 HPLGSTAMGGNNINFYSSPYLYNPKYGVRLGVEGKIDAVSVINLNAQKISLYN
UPAB1      541 HPLGSTAMGGNNINFYSSPYLYNPKYGVRLGVEGKIDAVSVINLNAQKISLYN
J15        541 HPLGSTAMGGNNINFYSSPYLYNPKYGVRLGVDGKIDAVSVINLNAQKISLYN
MRSN21681  541 HPLGSTAMGGNNINFYSSPYLYNPKYGVRLGVEGKIDAVSVINLNAQKISLYN
MRSN11669  541 HPLGSTAMGGNNINFYSSPYLYNPKYGVRLGVEGEIDAVSVINLNAQKISLYN
MRSN14237  541 HPLGSTAMGGNNINFYSSPYLYNPKYGVRLGVEGKIDAVSVINLNAQKISLYN
Ab04       541 HPLGSTAMGGNNINFYSSPYLYNPKYGVRLGEEGKIDAVSVINLNAQKISLYN
XH858      541 HPLGSTAMGGNNINFYSSPYLYNPKYGVRLGEEGKIDAVSVINLNAQKISLYN
NIPH_335   541 HPLGSTAMGGNNINFYSSPYLYNPKYGVRLGEEGKIDAVSVINLNAQKISLYN
OIFC098    541 HPLGSTAMGGNNINFYSSPYLYNPKYGVRLGEEGKIDAVSVINLNAQKISLYN
1419130    541 HPLGSTAMGGNNINFYSSPYLYNPKYGVRLGEEGKIDAVSVINLNAQKISLYN
NIPH_601   541 HPLGSTAMGGNNINFYSSPYLYNPKYGVRLGEEGKIDAVSVINLNAQKISLYN
consensus  541 HPLGSTAMGGNNINFYSSPYLYNPKYGVRLGeeGkIDAVSVINLNAQKISLYN
```
