# Supplementary material for: The Glycoprotease CpaA Secreted by Medically Relevant Acinetobacter Species Targets Multiple O-Linked Host Glycoproteins
Source: mBio. 2020 Oct 6;11(5):e02033-20. doi: 10.1128/mBio.02033-20 (PMC7542363; doi:10.1128/mBio.02033-20)
Supplement: TABLE S1 [file mBio.02033-20-st001.pdf]

| **Table S1. Strains, plasmids, and primers used in this study** | | |
|---|---|---|
| **Strain** | **Genotype** | **Reference/source** |
| Stellar | *F–, endA1, supE44, thi-1, recA1, relA1, gyrA96, phoA, Φ80d lacZΔ M15, Δ (lacZYA - argF) U169, Δ (mrr - hsdRMS - mcrBC), ΔmcrA, λ–* | Clontech |
| Δ*cpaAB* | *A. nosocomialis* M2 Δ *cpaAB::frt* | (1) |
| | | |
| **Plasmid** | **Description** | **Reference/source** |
| pWH-*cpaA-his-cpaB* | *cpaA-6xhis-cpaB* cloned into pWH1266 with its own promoter region | (2) |
| pMFH32 | CpaA E520A using pWH-*cpaA-his-cpaB* as template | (3) |
| pMFH44 | CpaA W493L using pWH-*cpaA-his-cpaB* as template | This study |
| pMFH45 | CpaA W493A using pWH-*cpaA-his-cpaB* as template | This study |
| pMFH46 | CpaA W493F using pWH-*cpaA-his-cpaB* as template | This study |
| | | |
| **Primer** | **Sequence** | |
| CpaA W493L 1 | aatccaagccaagccacaaccgccatcccc | |
| CpaA W493L 2 | ggggatggcggttgtggcttggcttggatt | |
| CpaA W493A 1 | agcattaatccaagccgcgccacaaccgccatcc | |
| CpaA W493A 2 | ggatggcggttgtggcgcggcttggattaatgct | |
| CpaA W493F 1 | gcattaatccaagcgaagccacaaccgccatcccc | |
| CpaA W493F 2 | ggggatggcggttgtggcttcgcttggattaatgc | |


1. Kinsella RL, Lopez J, Palmer LD, Salinas ND, Skaar EP, Tolia NH, Feldman MF. 2017. Defining the interaction of the protease CpaA with its type II secretion chaperone CpaB and its contribution to virulence in Acinetobacter species. J Biol Chem 292:19628–19638.

2. Harding CM, Kinsella RL, Palmer LD, Skaar EP, Feldman MF. 2016. Medically Relevant Acinetobacter Species Require a Type II Secretion System and Specific Membrane-Associated Chaperones for the Export of Multiple Substrates and Full Virulence. PLoS Pathog 12:e1005391.

3. Urusova D V., Kinsella RL, Salinas ND, Haurat MF, Feldman MF, Tolia NH. 2019. The structure of Acinetobacter-secreted protease CpaA complexed with its chaperone CpaB reveals a novel mode of a T2SS chaperone-substrate interaction. J Biol Chem 294:13344–13354.
