# Supplementary material for: The Glycoprotease CpaA Secreted by Medically Relevant Acinetobacter Species Targets Multiple O-Linked Host Glycoproteins
Source: mBio. 2020 Oct 6;11(5):e02033-20. doi: 10.1128/mBio.02033-20 (PMC7542363; doi:10.1128/mBio.02033-20)
Supplement: TABLE S2 [file mBio.02033-20-st002.pdf]

Table S2. Residues of the active site alpha helix used to overlay enzyme crystal structures. Conserved histidine residues are noted in bold.

**Enzyme**	**PDB Code**	**Helix Sequence**
CpaA	6O38.A	AAMR**H**EVG**H**N
Serralysin	3VI1.B	QTLT**H**EIG**H**T
IMPa	5KDX.B	WGES**H**ELG**H**N
ZmpB	5KDU.A	WGVA**H**ELG**H**N
ZmpB	5KDS.A	WGVA**H**ELG**H**N
BT4244	5KD8.A	WGPA**H**EIG**H**V
**The ligand and glycan of X-ray and modeled structures.** The first four entries are X-ray structures used to perform docking studies with the CpaA model and three glycopeptide substrates. The P and S residues of the consensus sequence are highlighted, with the asterisk denoting the point of glycosylation.		
**Protein**	**Peptide Ligand**	**Glycan Ligand**
Serralysin (3VI1.B)	R P K P Q Q	-
IMPa (5KDX.B)	-	Galβ1-3GalNAcα1-
ZmpB (5KDU.A)	-	Galβ1-3(Neu5Acα2-6)GalNAcα1-
Agglutinin (2CWG)	-	Neu5Acα2-3Galβ1-3GalNAcα1-
CpaA Model	Ac E A **P** **S*** A *N*Me	Galβ1-3GalNAcα1-
CpaA Model	Ac E A **P** **S*** A *N*Me	Galβ1-3(Neu5Acα2-6)GalNAcα1-
CpaA Model	Ac E A **P** **S*** A *N*Me	Neu5Acα2-3Galβ1-3GalNAcα1-
